# Deoxyfluorination with superacids – synthesis and characterization of protonated α-fluorohydroxyacetic acid†

**DOI:** 10.1039/d4ra05449k

**Published:** 2024-10-04

**Authors:** Alan Virmani, Christoph Jessen, Alexander Nitzer, Andreas J. Kornath

**Affiliations:** a Ludwig-Maximilians-Universität München Butenandtstraße 5-13 (D) D-81377 München Germany

## Abstract

α-Fluoroalcohols describe a rare and unstable class of compounds, accessible mainly by fluorination of highly electrophilic carbonyl compounds. In this work, we report the syntheses of α-fluorohydroxyacetic acid (FHA) and its acyl fluoride (FHA-F) by reacting the dihydroxy species glyoxylic acid monohydrate (GAM) with SF_4_. Surprisingly, only one of the geminal hydroxy groups is substituted when excess SF_4_ is employed. Implementing GAM with the binary superacid HF/AsF_5_ also leads to a single yet quantitative deoxyfluorination at the diol group. The reaction pathways are discussed based on NMR experiments, the characterization was carried out using NMR and vibrational spectroscopy as well as single-crystal X-ray diffraction.

## Abstract 2

In this work, we present an unconventional synthesis route to generate a novel α-fluorohydroxy compound with an additional functional group.

## Graphical abstract

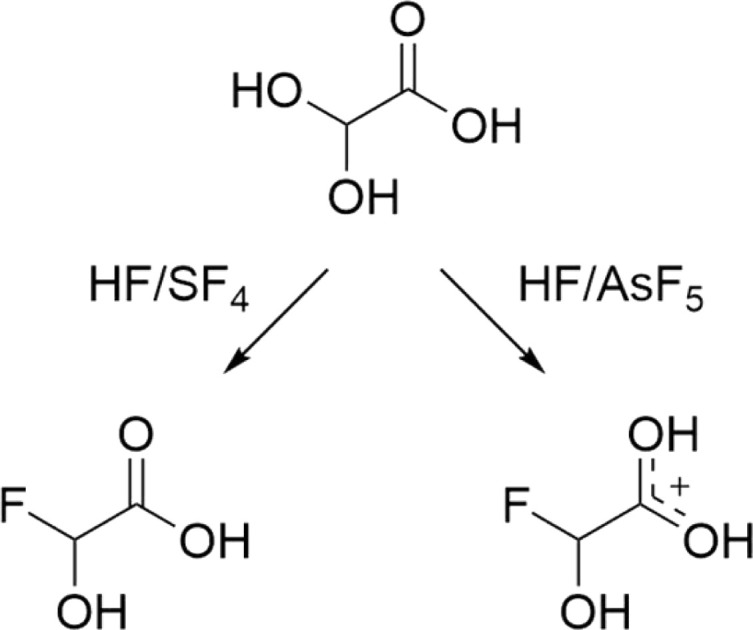

## Introduction

Organic compounds containing carbon atoms with more than one hydroxy group are known to be labile under regular conditions. According to the rule of Erlenmeyer, they undergo facile dehydration under the formation of the respective carbonyl compound. This also applies to alcohols with a geminal halogen atom, where the hydrogen halide is easily eliminated.^1^ In the case of fluorinated compounds, only a few examples of α-fluoroalcohols are known. Fluoromethanol CFH_2_OH, the simplest representative, was synthesized by Olah and Pavláth as early as 1953.^2^ In 1977, Seppelt was able to generate the perfluorinated alcohol trifluoromethanol CF_3_OH by reacting CF_3_OCl with HCl. He operated at low temperatures to prevent the decomposition under the formation of COF_2_ and HF, which is highly favored.^3^ 30 years later, Christe *et al.* investigated this equilibrium.^4^ The addition of HF or F^−^ to a carbonyl group is a convenient way to access (per-)fluorinated alcohols, first shown by Andreades and England in 1961,^5^ followed by others in recent studies.^6,7^ The general equation is given below (eqn (1)).1
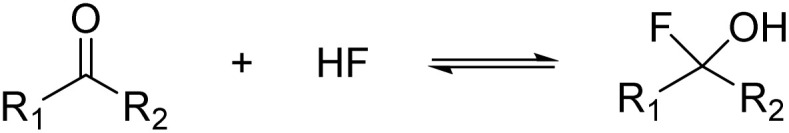


However, the α-fluoroalcohol is only stable when the electrophilicity of the carbonyl group is high enough, similar to the rule of Erlenmeyer.^8^ The equilibrium of eqn (1) can be shifted to the right by transforming the alcohol into stable derivatives like acetals or oxonium ions.^4,9,10^ The respective oxonium ions were generated by reacting the carbonyl compounds with the superacidic system HF/SbF_5_ in anhydrous hydrogen fluoride (aHF). In this way, the perfluorinated oxonium ions of methanol, ethanol, *n*-propanol,^10^ and isopropanol^11^ have been synthesized.

An example of an exception to the rule of Erlenmeyer is glyoxylic acid (GA). The purchasable monohydrate form (GAM) does not imply co-crystallized but chemically bound water and is better described as dihydroxyacetic acid. Its reactivity toward highly acidic systems, in which it can be activated for electrophilic reactions, has been described by Prakash *et al.*^12^ The high electrophilicity makes it an interesting target for generating α-fluorohydroxy compounds with an additional functional group in the direct vicinity. To exploit this possibility or to determine if a difluorinated product is formed, we have implemented GAM with the deoxyfluorinating agent SF_4_ as well as the superacidic medium HF/AsF_5_. We wish to report the results herein.

## Results and discussion

### Syntheses and properties

α-Fluorohydroxyacetic acid (FHA, 1) is synthesized by reacting glyoxylic acid monohydrate (GAM) with an equimolar amount of sulfur tetrafluoride (eqn (2)). For the synthesis of α-fluorohydroxyacetyl fluoride (FHA-F, 2), a twofold amount of SF_4_ is applied (eqn (3)). The formation of difluoroacetyl fluoride was not observed with an excess of SF_4_.2
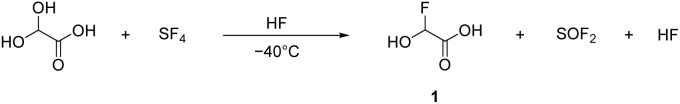
3
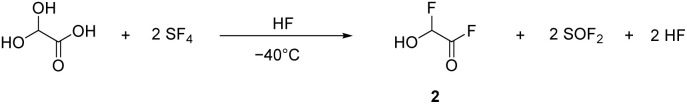


The mechanism of GAM in the system HF/SF_4_ is proposed based on the literature-reported pathways of similar reactions.^13–15^ In the first step, SF_4_ dissociates in aHF according to eqn (4).4
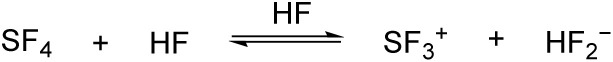


**Scheme 1:**
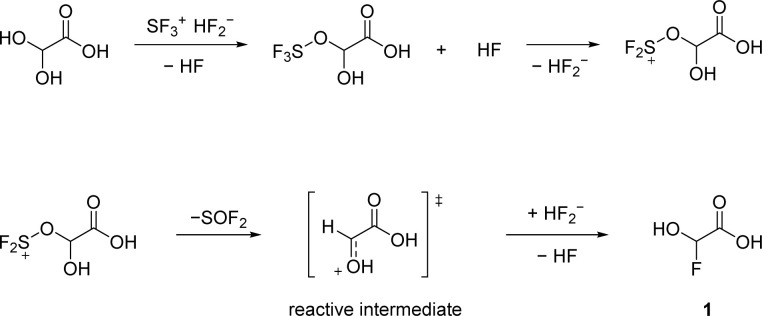
Proposed reaction pathway of GAM with equimolar amounts of SF_4_.

We confirm that the alcohol moiety is more nucleophilic than the carboxy group, which is why the first deoxyfluorination takes place there (Scheme 1). The reactive intermediate is a planar oxonium ion. Since the addition of a nucleophile in this mechanism is not stereoselective, a racemic mixture is expected.

**Scheme 2:**
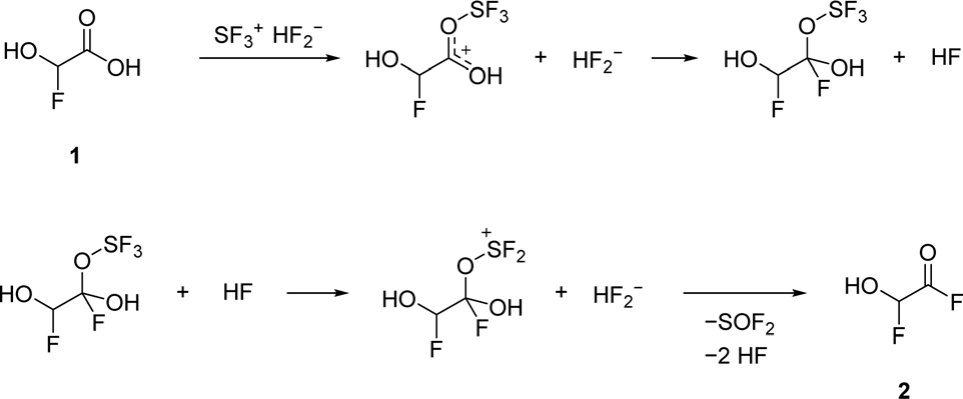
Proposed reaction pathway of the deoxyfluorination reaction at the carboxy group.

The second deoxyfluorination of the carboxylic group proceeds in a similar fashion. However, in this case, the formation of a tetrahedral intermediate is likely, as it has been suggested in previous studies about the reactions of carbonyl compounds with HF/SF_4_.^13,15^ The proposed mechanism is illustrated in Scheme 2.

Employing three or more equivalents of SF_4_ did not result in a third deoxyfluorination of the last hydroxy group, hence in 2,2-difluoroacetyl fluoride. Since the cationic intermediate would be a fluoro carbenium ion, it is presumably not sufficiently stabilized.

The deoxyfluorination agent SF_4_ has been investigated in the past.^13,15^ Interestingly, a similar reaction is observed for GAM reacting with superacids. Protonated α-fluorohydroxyacetic acid [FHA-1H]^+^ is generated from GAM in the superacidic system HF/AsF_5_, resulting in [FHA-1H][AsF_6_] (3). The reaction is visualized in eqn (5).5
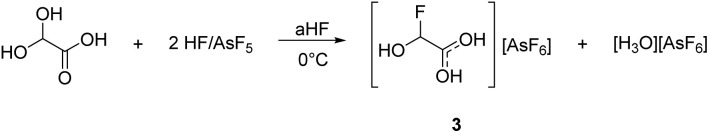


**Scheme 3:**
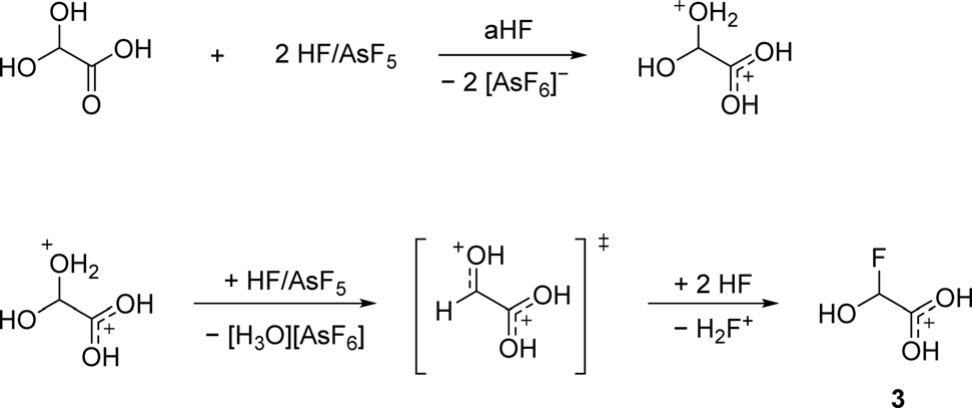
Proposed mechanism of the synthesis of [FHA-1H]^+^ from GAM.

According to eqn (5), a two-to-one ratio of AsF_5_ to GAM would formally suffice to form [FHA-1H][AsF_6_]. However, full conversion takes place only when three equivalents of Lewis acid are applied. The carboxy group is more basic than the hydroxy groups due to better resonance stabilization, which is why it is likely protonated in the first step. The second protonation occurs at one of the hydroxy groups that subsequently is eliminated as H_3_O^+^. The formed substituted ethylene dication [C_2_(OH)_3_H]^2+^ is superelectrophilic enough to add fluoride from the solvent aHF, similar to observations we made in a recent study.^16^3 is found as a racemic mixture of the two enantiomers, strongly indicating an S_N_1 mechanism. The proposed mechanism is displayed in Scheme 3.

The necessity of three equivalents of Lewis acid to form 3 leads to the conclusion that a superelectrophilic carbodication is formed as the reactive intermediate.

Prakash *et al.*^12^ showed that the carbonyl group (or dihydroxy group, respectively) of glyoxylic acid can be activated with strong acids in the presence of aromatic compounds for electrophilic reactions to synthesize diarylacetic acid derivatives (eqn (6)). α-Fluorohydroxyacetic acid may be useful similarly to generate aryl-fluoroacetyl acid derivatives (eqn (7)), while α-fluorohydroxyacetyl fluoride has the potential to be activated with Lewis acids at the C1-atom for Friedel-Crafts-acylations (eqn (8)). However, for this kind of reaction, the protonated species of α-fluorohydroxyacetic acid might be a better fit since the carboxy group is already activated and its [AsF_6_]^−^ salt is relatively stable (eqn (9)).6
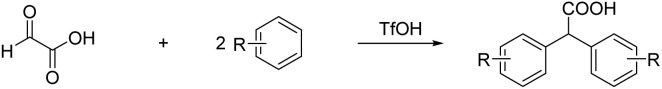
7
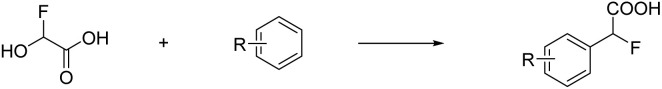
8
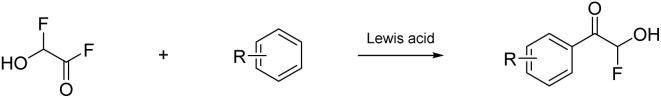
9
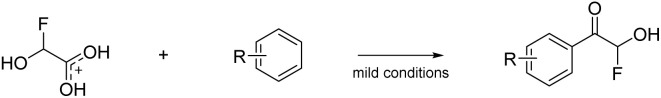


### NMR spectroscopy

The reactivity of glyoxylic acid monohydrate (GAM) in the systems HF/SF_4_, HF/AsF_5_, or solely aHF can be traced by ^1^H, ^19^F, and ^13^C NMR spectroscopy. The samples were dissolved either in aHF or SO_2_, and acetone-*d*_6_ was employed for external referencing. For more details of the experimental procedure, see the ESI† (Apparatus and Materials). The chemical shifts of 1, 2, and 3 are listed in Table 1. The respective solvent used for the measurements is given in the table footnote. A reference of GAM in D_2_O is displayed in Fig. S1 and S2.†

**Table:** ^1^H, ^19^F, and ^13^C chemical shifts [ppm] including coupling constants [Hz] of FHA (1), FHA-F (2), and [FHA-1H][AsF_6_] (3). Measured at −40 °C

	FHA^a^	FHA-F^b^	[FHA-1H][AsF_6_]^a^
*δ* [^1^H] (C–H)	5.57 (d), *J* = 54.4	7.00 (d) *J* = 51.2	5.61 (d) *J* = 56.9
*δ* [^1^H] (H_3_O^+^)			9.48 (s)
*δ* [^19^F] (C–F)	−130.38 (d) *J* = 54.4	−134.67 (d) *J* = 53.7	−128.88 (d) *J* = 53.9
*δ* [^19^F] (COF)		23.63 (d) *J* = 16.3	
		22.97 (d) *J* = 14.1	
*δ* [^13^C] (carboxylic)	170.98 (d) *J* = 32.7	154.33 (dd) *J* = 368.7, 34.8	184.90 (d) *J* = 34.0
*δ* [^13^C] (tetrahedral)	96.95 (d) *J* = 225.1	92.59 (dd) *J* = 241.3, 82.4	97.00 (d) *J* = 227.1

a aHF as a solvent.

b SO_2_ as a solvent.

GAM has proven to be very reactive to HF. When dissolved in aHF, NMR spectra (Fig. S3–S5, ESI†) show a variety of fluorinated compounds. Doublets in both the ^1^H (5.65 ppm, *J* = 61.2 Hz) and the ^13^C NMR spectra (171.57 ppm, *J* = 32.6 Hz and 97.99 ppm, *J* = 225.2 Hz) are very similar to those assigned to 1. This means that deoxyfluorination occurs in aHF, albeit uncontrolled. By first dissolving equimolar amounts of SF_4_ (compared to GAM) in aHF and secondly adding GAM, 1 becomes the main product with an amount of roughly 74% (NMR spectra displayed in Fig. S6–S8, ESI†), as the doublet at 5.57 ppm is the most intensive one in the ^1^H NMR spectrum. The ^19^F signal at −130.38 ppm is assigned to the fluorine atom in FHA since the coupling constant is the same as for the ^1^H signal. The ^13^C shift of the carboxy group is observed at 170.98 ppm, and the tetrahedral carbon shift at 96.95 ppm.

By employing two equivalents of SF_4_, 2 is the most abundant species. The NMR spectra recorded in aHF showed an equilibrium of 2 and its HF-adduct 4 (see eqn (10)). The spectra are displayed in Fig. S9–S11 in the ESI.†10
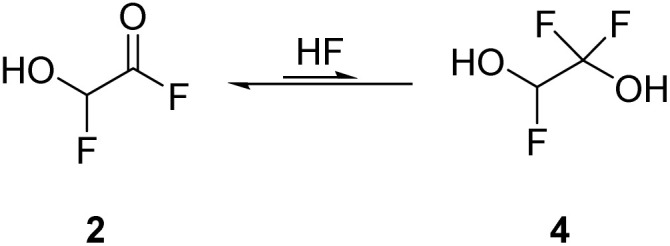


The equilibrium is shifted to 2 by removing all volatile products at −78 °C, successively warming up the residue to 0 °C, and trapping the gas phase into a second vessel at −196 °C. The condensate was dissolved in SO_2_, and NMR spectra (Fig. S12–S14†) show 2 as the only organic compound, as shown by the intensive doublet at 7.00 ppm in the ^1^H NMR spectrum. The ^13^C signal of the acyl fluoride is observed at 154.33 ppm. The dd-splitting pattern shows the coupling to two fluorine atoms, just like the ^13^C shift of the tetrahedral carbon at 92.59 ppm. In the ^19^F NMR spectrum, these signals occur at −134.67, 23.63, and 22.97 ppm. Additional signals at 65.17 ppm and 64.48 ppm are assigned to residual SF_4_ that has not been removed.^17^

A different approach to generating a derivative of α-fluorohydroxyacetic acid is the deoxyfluorination of GAM with superacids. By dissolving the Lewis acid in aHF in the first step, the superacidic medium is formed. Subsequently adding GAM to the solution led to a quantitative synthesis of [FHA-1H][AsF_6_] (3) (NMR spectra displayed in Fig. S15–S17, ESI†). The ^13^C signal of the carboxy group (184.90 ppm) is shifted downfield compared to GAM and 1 due to the protonation in the superacidic system, similar to reported protonated carboxy groups.^18^ The doublet at 97.00 ppm is assigned to the CHF(OH) group. The ^19^F signal is observed at −128.88 ppm and the ^1^H resonance at 5.61 ppm. The ^1^H signal at 9.48 ppm confirms the formation of H_3_O^+^.^19^

### Vibrational spectroscopy

**Fig. 1:**
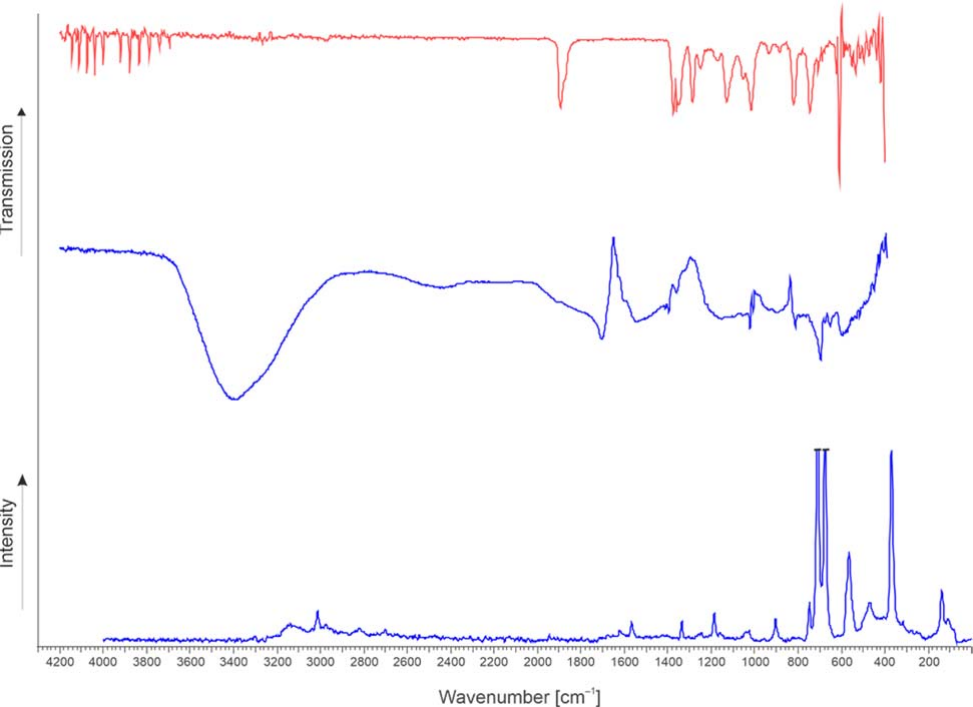
The infrared spectrum of gaseous 2 at room temperature (top, red). Low-temperature infrared (middle) and Raman spectrum (bottom) of 3 (blue).

The synthesis of FHA-F and [FHA-1H][AsF_6_] is confirmed by vibrational spectroscopy. FHA-F (2) was generated by reacting GAM with a twofold amount of SF_4_ in aHF. The solvent and other volatile products were removed *in vacuo* overnight at −78 °C. The sample was warmed up to 0 °C and an infrared spectrum of the gas phase was measured at room temperature. In the case of [FHA-1H]^+^, the [AsF_6_]^−^ salt (3) was synthesized by reacting GAM with HF/AsF_5_ in aHF and successively removing the solvent *in vacuo* at −78 °C. Infrared and Raman spectroscopy of the colorless residue was performed at low temperatures (see ESI† for more details). The spectra are displayed in Fig. 1 and selected observed frequencies are listed in Table 2. Quantum chemical calculations of the respective compounds were performed to support the assignment of the vibrational frequencies. The detailed characterization of the compounds is found in the ESI.† FHA (1) could not be isolated from aHF, so an experimental frequency analysis was not feasible. However, the quantum chemically calculated frequencies and their assignment are listed in Table S3 in the ESI.†

**Table:** Selected observed vibrational frequencies [cm^−1^] of 2 and 3

FHA-F	[FHA-1H][AsF_6_]		Assignment
Exp. IR^a^	Exp. IR^a^	Exp. Ra^b^	
1894 (m)	1705 (m)	1567 (7)	*ν*(CO_a_)
	1541 (m)		*ν*(CO_a_)
1173 (m)			*ν*(C(O)F)
1128 (m)	1151 (m)	1162 (3)	*ν*(C–OH)
1016 (m)	1022 (m)	1027 (4)	*ν*(CF)
822 (m)	897 (m)	903 (8)	*ν*(CC)

a Abbreviations: m = medium, a = acid.

b Experimental Raman intensities are relative to a scale of 1 to 100.

The infrared spectrum of 2 shows rotational bands of remaining hydrogen fluoride between 3728 and 4143 cm^−1^, which was not completely removed after the reaction. The *ν*(C=O) vibration is observed at 1894 cm^−1^. This band is distinct for acyl fluorides^20–22^ and is significantly blue-shifted compared to GAM (1742 cm^−1^).^23^ The stretching vibrations of the newly formed CF bonds occur at 1173 (acyl fluoride moiety) and 1016 cm^−1^ (fluorohydroxy group). The intensive bands down from 708 cm^−1^ are assigned to residual SF_4_, which has been observed in the NMR study as well. A reference spectrum of SF_4_ is illustrated in Fig. S18 in the ESI.†

In the IR spectrum of 3, a strong and broad band with a maximum at 3406 cm^−1^ is found. This might be assigned to H_3_O^+^, however, it cannot be excluded that this band is attributed to the measurement method at low temperatures, where water can condense onto the specimen, superposing the OH and CH stretching vibrations. The protonation of the carboxy group can be traced by the *ν*_as_(CO) band at 1705 cm^−1^ (IR), which is red-shifted compared to *ν*(C=O) of GAM (1742 cm^−1^).^23^ The antisymmetric CO stretching mode of 3 occurs at 1541 (IR) and 1567 cm^−1^ and is in return blue-shifted concerning *ν*(C–O) of GAM (1101 cm^−1^). This convergence of the carboxylic vibrations is a direct result of protonation and has been described in several studies.^24,25^ The stretching vibration of the newly formed CF bond is observed at 1022 (IR) and 1027 cm^−1^ (Ra), similar to 2.

### Crystal structure of [FHA-1H][AsF_6_]

**Fig. 2:**
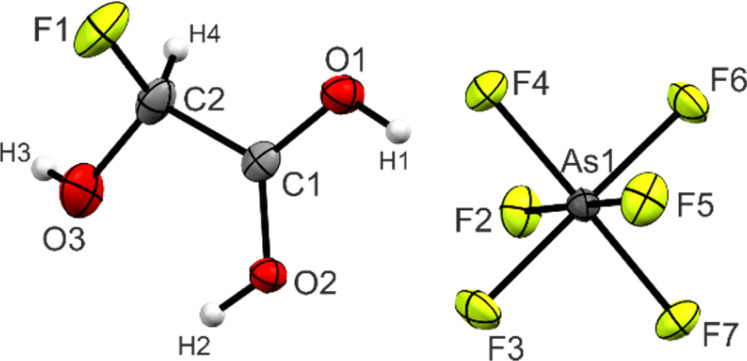
Projection of the asymmetric unit of 3 (50% probability displacement ellipsoids, hydrogen atoms displayed as spheres of arbitrary radius).

Single crystals of 3 were obtained by dissolving the colorless powder in aHF at −55 °C. Colorless needles suitable for single-crystal X-ray diffraction grew as racemic twins within three days. In the following, the S-enantiomer is discussed. 3 crystallizes in the orthorhombic space group *P*2_1_2_1_2_1_ with four formula units per unit cell. The asymmetric unit is displayed in Fig. 2. Table 3 contains selected geometric parameters.

Selected geometric parameters of 3. Symmetry codes: *i* = *x*, 1 + *y*, *z*; *ii* = 1.5 − *x*, 2 − *y*, 0.5 + *z*

**Table:** 

Bond lengths [Å]		Intermolecular interactions D(–H)⋯A [Å]	
C1–O1	1.258(4)	O1(–H1)⋯F2	2.579(4)
C1–O2	1.272(4)	O2(–H2)⋯O3	2.587(4)
C1–C2	1.515(5)	O2(–H2)⋯F6*i*	2.669(3)
C2–O3	1.355(5)	O3(–H3)⋯F3*ii*	2.826(4)
C2–F1	1.376(5)	C1⋯F7	2.733(5)

**Table:** 

Bond angles [deg]		Dihedral angles [deg]	
O1–C1–O2	120.6(3)	O3–C2–C1–O1	179.5(4)
O1–C1–C2	117.4(3)	F1–C2–C1–O1	−61.2(5)
O2–C1–C2	122.0(3)	O3–C2–C1–O2	−1.0(6)
O3–C2–C1	106.6(3)	F1–C2–C1–O2	118.3(4)
F1–C2–C1	106.1(4)		
O3–C2–F1	111.9(4)		

The C1–C2 bond of 1.515(5) Å is similar to the starting material glyoxylic acid monohydrate (GAM, 1.522(3) Å),^26^ yet slightly longer than a regular Csp^3^–Csp^2^ single bond (1.502 Å) in carboxylic acids.^27^ The C1–O1 (1.258(4) Å) and C1–O2 (1.272(4) Å) bond distances are approximately the same, as has been observed in a variety of protonated carboxylic acids.^24,25,28^ The C2–O3 bond (1.355(5) Å) is significantly shorter than the two C–OH bonds in GAM (1.400(4) and 1.404(3) Å) and even more significant than a regular C–O single bond in primary alcohols (1.426 Å).^27^ The newly formed C2–F1 of 1.376(5) Å, on the other hand, is longer than a regular C–F bond with an electron-withdrawing group in the geminal position (1.349 Å).^27^ The nature of this bond relation will be discussed below in the Theoretical Study.

**Fig. 3:**
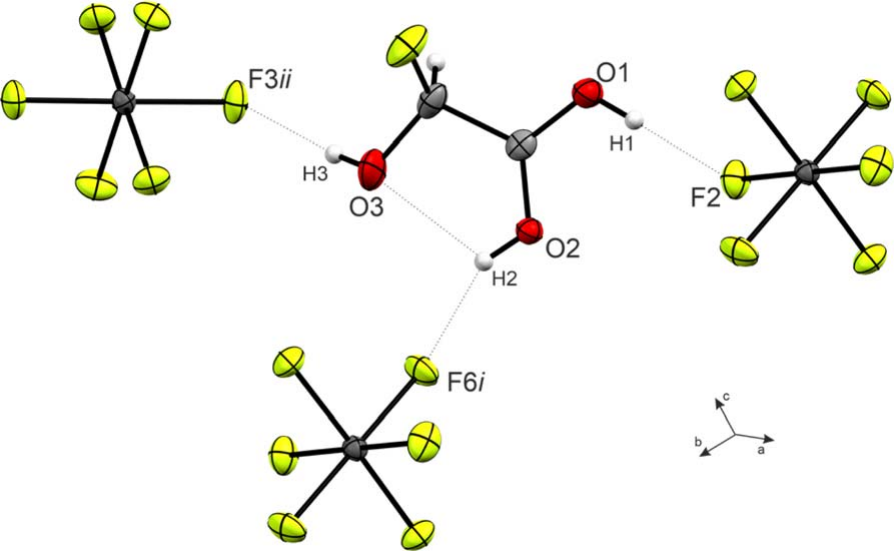
Hydrogen bonds in the crystal packing of 3 (50% probability displacement ellipsoids, hydrogen atoms displayed as spheres of arbitrary radius).

The O1–C1–O2 angle of 120.6(3)° is significantly smaller than in GAM (125.1(2)°)^26^ as a result of the protonated carboxy group. Subsequently, the O1–C1–C2 is widened from 111.9(2)° in GAM to 117.4(3)° in 3, while the remaining bond angles remain approximately unchanged. Regarding the torsion angles, the O3–C2–C1–O2 dihedral is reduced from 9.9(9)° to −1.0(6)°. This is due to an intramolecular hydrogen bond O2(–H2)⋯O3 with a distance of 2.587(4) Å that is formed upon protonation. The cation exhibits three additional, moderately strong hydrogen bonds^29^ (Fig. 3) to form layers in the *bc*-plane (O1(–H1)⋯F2, O2(–H2)⋯F6, and O3(–H3)⋯F3). These layers are connected along the *a*-axis by nearly perpendicular C1⋯F7 interactions with a distance of 2.733(5) Å (Fig. S21, ESI†), which is about 14% within the sum of the van-der-Waals radii (3.17 Å).^30^

The bond distances of the anion range between 1.698(3) and 1.754(2) Å. As–F bonds involved in donor–acceptor interactions (As1–F2, As1–F2, and As1–F6) are slightly longer than the others, resulting in a distorted octahedral structure. These values have been observed for [AsF_6_]^−^ anions in literature.^28,31,32^

### Theoretical Study

**Fig. 4:**
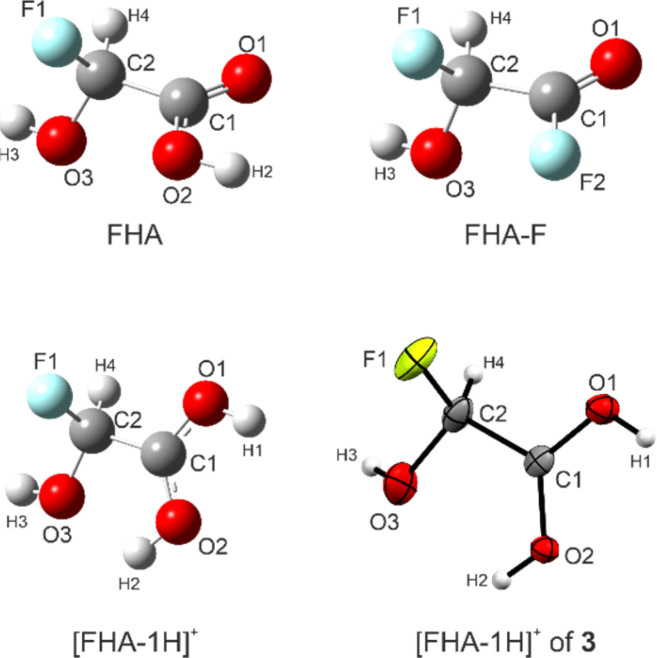
Optimized gas-phase structures of FHA, FHA-F, [FHA-1H]^+^, and the cation [FHA-1H]^+^ of 3. Calculated on the B3LYP/aug-cc-pVTZ level of theory.

For FHA (1), FHA-F (2), and the free [FHA-1H]^+^ cation, quantum chemical calculations were performed. The gas-phase structures were optimized and the vibrational frequencies were computed on the B3LYP/aug-cc-pVTZ level of theory. In the case of the cation, a direct comparison to the experimental X-ray values is possible. The bond lengths are listed in Table 4. The calculated structures are illustrated together with the cation of 3 in Fig. 4. The labeling of the atoms is based on the crystal structure analysis for consistency.

**Table:** Calculated bond distances [Å] of FHA, FHA-F, and [FHA-1H]^+^ compared to the experimental values of 3 obtained from the X-ray structure analysis

	FHA^a^	FHA-F^a^	[FHA-1H]^+^^a^	X-ray (3)
C1–O1	1.201	1.180	1.268	1.258(4)
C1–O2/F2	1.338	1.342	1.268	1.272(4)
C1–C2	1.532	1.529	1.538	1.515(5)
C2–O3	1.376	1.373	1.368	1.355(5)
C2–F1	1.392	1.389	1.367	1.376(5)

a Calculated on the B3LYP/aug-cc-pVTZ level of theory.

The C–O bond distances of the protonated carboxy group are in fair agreement with the experimental values. For FHA, the values are consistent with comparable carboxylic acids like GAM or difluoroacetic acid.^26,33^ The C=O bond length of FHA-F is the shortest among the investigated, yet it is in agreement with structural analyses of gaseous acyl fluorides reported in the literature, as well as the C(O)F bond.^34,35^ The C2–O3 distances of all calculated structures (1.368–1.376 Å) are similar, while the X-ray data of 3 is a little shorter. However, all these values are shorter than a regular C–O single bond in primary alcohols (1.426 Å).^27^ The calculated C2–F1 bond lengths of FHA and FHA-F are longer than in the case of [FHA-1H]^+^. The bond distances of α-fluoroalcohols compared to regular C–F and C–OH bonds have been discussed in a study by Krossing *et al.* Accordingly, the elongation of the C–F bond is suspected to be a result of lone-pair conjugation of the oxygen atom into the antibonding σ*(C–F) orbital, subsequently shortening the C–OH bond.^7^ This is in agreement with our DFT results. We performed NBO calculations of all three investigated compounds (MP2/aug-cc-pVTZ level of theory) to assess this effect. The stabilization energies according to the second-order perturbation theory analyses of these interactions are summarized in Table 5.

**Table:** The stabilization energy by the non-bonding lone-pairs (*n*) of the O3 and the F1 atom. NBO calculations on the MP2/aug-cc-pVTZ level of theory^a^

	*n*(O3) → σ*(C2–F1)	*n*(F1) → σ*(C1–C2)
FHA	109.3 kJ mol^−1^	16.8 kJ mol^−1^
FHA-F	111.5 kJ mol^−1^	19.3 kJ mol^−1^
[FHA-1H]^+^	105.7 kJ mol^−1^	29.0 kJ mol^−1^

a Calculated on the MP2/aug-cc-pVTZ level of theory.

The interactions of the oxygen lone-pair with the σ*(C–F) orbital are similar among the investigated compounds, explaining the shortening of the C–OH bond. The C–F distance of the neutral compounds FHA and FHA-F is subsequently elongated. In the case of the protonated species, the calculated C–F bond length rather coincides with a regular distance. Since the protonation has no significant influence on the described interaction, there must be another that strengthens the C–F bond. This is found to be the donation of a fluorine lone-pair into the σ*(C–C) orbital. The stabilization energy of this interaction in [FHA-1H]^+^ is calculated to be 12.2 kJ mol^−1^ higher than in FHA. This also explains why the C–C bond of the protonated species is the longest. However, the calculation estimates it longer than the experimental X-ray data shows. Similarly, the C–C bonds of FHA (1.532 Å) and FHA-F (1.529 Å) are longer than expected when compared to the corresponding bonds in difluoroacetic acid and difluoroacetyl fluoride.^35^ This indicates that solid-state effects might have an additional influence on the bond distances.

## Conclusions

For the first time, α-fluorohydroxyacetic acid (FHA), its acyl fluoride (FHA-F), and its protonated species ([FHA-1H]^+^) are described. The syntheses of FHA and FHA-F are achieved by reacting glyoxylic acid monohydrate (GAM) with HF/SF_4_. By applying the binary superacid HF/AsF_5_, [FHA-1H][AsF_6_] is the only organic compound, allowing a complete characterization by NMR, vibrational spectroscopy, and single-crystal X-ray diffraction. The superacidic deoxyfluorination only occurs when three equivalents of Lewis acid are used, implying that the superelectrophile [C_2_(OH)_3_H]^2+^ is formed intermediately. NBO calculations reveal a complex relation between the C–F and the C–OH bond of the fluorohydroxy group. The use of superacids in aHF could enable convenient access to fluorinated compounds with a high electrophilicity and give deoxyfluorination reagents a new appeal.

## Data availability

The data supporting this article have been included as part of the ESI.† For full details on vibrational spectroscopy, NMR spectroscopy, X-ray diffraction refinement, and computational details see the ESI.† Crystallographic data for [CH(OH)FC(OH)_2_][AsF_6_] has been deposited at the CCDC under the accession number 2173682 and can be obtained from http://www.ccdc.cam.ac.uk.

## Conflicts of interest

There are no conflicts to declare.

## Supplementary Material

RA-014-D4RA05449K-s001

RA-014-D4RA05449K-s002
