# Supplementary material for: Deoxyfluorination with superacids – synthesis and characterization of protonated α-fluorohydroxyacetic acid
Source: RSC Adv. 2024 Oct 4;14(43):31517–23. doi: 10.1039/d4ra05449k (PMC11450550; doi:10.1039/d4ra05449k)
Supplement: RA-014-D4RA05449K-s001 [file RA-014-D4RA05449K-s001.pdf]

Supplementary Information (SI) for RSC Advances.
This journal is © The Royal Society of Chemistry 2024

Supporting Information

Deoxyfluorination with Superacids – Synthesis and Characterization of Protonated α-Fluorohydroxyacetic Acid

Alan Virmani, Alexander Nitzer, Christoph Jessen, and Andreas J. Kornath*

Abstract: α-Fluoroalcohols are a rare and unstable class of compounds, accessible mainly by fluorination of highly electrophilic carbonyl compounds. In this work, we report the syntheses of α-fluorohydroxyacetic acid (FHA) and its acyl fluoride (FHA-F) by reacting the dihydroxy species glyoxylic acid monohydrate (GAM) with SF_4. Surprisingly, only one of the geminal hydroxy groups is substituted when excess SF_4 is employed. Implementing GAM with the binary superacids HF/MF_5 (M = As, Sb) also leads to a single yet quantitative deoxyfluorination at the diol group. The reaction pathways are elucidated by NMR experiments, the characterization was carried out using NMR and vibrational spectroscopy as well as single-crystal X-ray diffraction.

Table of Contents

Experimental Procedures....4

Apparatus and Materials....4

Synthesis of α-Fluorohydroxyacetic Acid....5

Synthesis of α-Fluorohydroxyacetic Fluoride....5

Synthesis of Protonated α-Fluorohydroxyacetic Acid....5

Results and Discussion....5

NMR Spectroscopy....5

Glyoxylic Acid Monohydrate (GAM) in D_2O....5

Glyoxylic Acid Monohydrate (GAM) dissolved in aHF....7

2,2-Fluorohydroxyacetic Acid (**1**)....9

2,2-Fluorohydroxyacetyl Fluoride (**2**)....11

Protonated α-Fluorohydroxyacetic Acid [FHA-1H][AsF_6] (**3**)....14

Vibrational Spectroscopy....17

Single-Crystal X-ray Structure Analysis....19

Crystal Structure of [FHA-1H][AsF_6]....19

Theoretical Study....23

α-Fluorohydroxyacetic Acid (FHA)....23

α-Fluorohydroxyacetic Acid (FHA-F)....23

Protonated α-Fluorohydroxyacetic Acid ([FHA-1H]$^+$)....24

References....25

List of Figures

Figure S1. ^1H NMR spectrum of GAM in D_2O at room temperature. 6

Figure S2. ^{13}C {^1H} NMR spectrum of GAM in D_2O at room temperature. 6

Figure S3. ^1H NMR spectrum of GAM in aHF at 0°C. 7

Figure S4. ^{19}F NMR spectrum of GAM in aHF at 0°C. 8

Figure S5. ^{13}C NMR spectrum of GAM in aHF at 0°C. 8

Figure S6. ^1H NMR spectrum of GAM with equimolar amounts of SF_4 in aHF at −40°C. 9

Figure S7. ^{19}F NMR spectrum of GAM with equimolar amounts of SF_4 in aHF at −40°C. 10

Figure S8. ^{13}C {^1H} NMR spectrum of GAM with equimolar amounts of SF_4 in aHF at −40°C. 10

Figure S9. ^1H NMR spectrum of GAM with a twofold amount of SF_4 in aHF at −40°C. 11

Figure S10. ^{19}F NMR spectrum of GAM with a twofold amount of SF_4 in aHF at −40°C. 12

Figure S11. ^{13}C {^1H} NMR spectrum of GAM with a twofold amount of SF_4 in aHF at −40°C. 12

Figure S12. ^1H NMR spectrum of **2** in SO_2 at −40°C. 13

Figure S13. ^{19}F NMR spectrum of **2** in SO_2 at −40°C. 13

Figure S14. ^{13}C {^1H} NMR spectrum of **2** in SO_2 at −40°C. 14

Figure S15. ^1H NMR spectrum of **3** in aHF at −40°C. 15

Figure S16. ^{19}F NMR spectrum of **3** in aHF at −40°C. 15

Figure S17. ^{13}C {^1H} NMR spectrum of **3** in aHF at −40°C. 16

Figure S18. Reference spectrum of gaseous SF_4. 18

Figure S19. Projection of the asymmetric unit of **3**. Thermal ellipsoid displacement probability set at 50%, hydrogen atoms displayed as spheres of arbitrary radius. 19

Figure S20. Hydrogen bonds of the cation in the crystal packing of **3**. Thermal ellipsoid displacement probability set at 50%, hydrogen atoms displayed as spheres of arbitrary radius. 20

Figure S21. Non-hydrogen bonded cation-anion interaction in the crystal packing of **3**. Thermal ellipsoid displacement probability set at 50%, hydrogen atoms displayed as spheres of arbitrary radius. 20

Figure S22. Crystal packing of **3** with a view along the *b*-axis. Thermal ellipsoid displacement probability set at 50%, hydrogen atoms displayed as spheres of arbitrary radius. 20

Figure S23. Crystal packing of **3**. Thermal ellipsoid displacement probability set at 50%, hydrogen atoms displayed as spheres of arbitrary radius. 21

Figure S24. Optimization of the gas-phase structure of FHA. Calculated on the B3LYP/aug-cc-pVTZ level of theory. 23

Figure S25. Optimization of the gas-phase structure of FHA-F. Calculated on the B3LYP/aug-cc-pVTZ level of theory. 23

Figure S26. Optimization of the gas-phase structure of $[FHA\text{-}1H]^+$. Calculated on the B3LYP/aug-cc-pVTZ level of theory. 24

List of Tables

Table S1. Experimental vibrational frequencies [cm^{-1}] of [FHA-1H][AsF_6] (**3**) and calculated frequencies of $[FHA\text{-}1H]^+$ (B3LYP/aug-cc-pVTZ level of theory). 17

Table S2. Experimental and calculated IR frequencies [cm^{-1}] of FHA-F (**2**). Calculated on the B3LYP/aug-cc-pVTZ level of theory. 18

Table S3. Quantum chemically calculated vibrational frequencies of **1**. Calculated on the B3LYP/aug-cc-pVTZ level of theory. 19

Table S4. Bond lengths [Å], bond angles, and torsion angles [°] of **3**. 21

Table S5. Summary of the X-ray diffraction data collection and refinement. 22

Table S6: Standard orientation of FHA. Calculated on the B3LYP/aug-cc-pVTZ level of theory. 23

Table S7. Standard orientation of FHA-F. Calculated on the B3LYP/aug-cc-pVTZ level of theory. 23

Table S8. Standard orientation of $[FHA\text{-}1H]^+$. Calculated on the B3LYP/aug-cc-pVTZ level of theory. 24

Experimental Procedures

Caution! Avoid contact with any of these materials. Hydrogen fluoride will be formed by the hydrolysis of these compounds. HF burns the skin and causes irreparable damage. Safety precautions should be taken when using and handling these materials.

Apparatus and Materials

All reactions were carried out at standard Schlenk conditions by using sealed 6 mm FEP/PFA reactors closed with a stainless-steel valve and a stainless-steel vacuum line. All vessels have been dried with fluorine prior to each reaction or NMR measurement. Glyoxylic acid monohydrate (abcr) was stored under a nitrogen atmosphere. SF_4 (abcr) was used as purchased, and antimony pentafluoride (VWR) was distilled three times prior to use. Arsenic pentafluoride was synthesized from the elements and purified by fractionated distillation.

Raman spectra were rendered with a Bruker MultiRAM FT-Raman spectrometer with an Nd:YAG laser excitation up to 1000 mW (λ = 1064 nm) in a usable range between 200 cm^{-1} and 4000 cm^{-1}. A measurement was performed after transferring the sample into a cooled (−196°C) glass cell under a nitrogen atmosphere and subsequent evacuation of the glass cell.

Low-temperature IR-spectroscopic investigations were carried out with a Bruker Vertex-80V FTIR spectrometer using a cooled cell with a single-crystal CsBr plate on which small amounts of the samples were placed.[1]

Single-crystal X-ray structure investigations were carried out with an Oxford Xcalibur3 diffractometer equipped with a Spellman generator (50 kV, 40 mA) and a KappaCCD detector. The measurements were performed with Mo-K_α radiation (λ = 0.71073 Å). For data collection, the software CrysAlis CCD,[2] for data reduction the software CrysAlis RED[3] was used. The solution and refinement were performed with the programs SHELXT[4] and SHELXL-97[5] implemented in the WinGX software package[6] and checked with the software PLATON.[7] The absorption correction was achieved with the SCALE3 ABSPACK multi-scan method.[8]

Quantum chemical calculations were performed with the Gaussian 16 program package.[9] Calculations were carried out employing the method B3LYP and the basis sets aug-cc-pVTZ. For visualization of the structures and vibrational modes, the program GaussView 6.0[10] was employed.

NMR spectra were recorded either on a Jeol ECX400 NMR or a Bruker AV400 NMR instrument. For visualization and evaluation, the software MestReNova Version 14.0.0 was used.[11] The spectrometers were externally referenced to $CFCl_3$ for ^{19}F and to tetramethylsilane for ^{1}H and ^{13}C NMR spectra. NMR measurements were prepared by dissolving the sample in aHF or SO_2 at the respective temperature and subsequently transferring the solution under a nitrogen atmosphere into a sealed 4 mm FEP tube at −78°C. After cooling down to −196°C the FEP tube was flame-sealed *in vacuo*. Immediately before the NMR measurement, the sealed FEP tube was put in a standard glass NMR tube loaded with 0.2 mL acetone-d_6 as an external reference and warmed to the designated temperature.

Synthesis of **α**-Fluorohydroxyacetic Acid

First, the FEP reactor was dried chemically with F_2. Sulfur tetrafluoride (108 mg, 1.00 mmol) and anhydrous hydrogen fluoride (approximately 0.5 mL) were then successively condensed into the reactions vessel at −196°C and agitated at −40°C. After cooling down to −196°C, glyoxylic acid monohydrate (92.05 mg, 1.00 mmol) was added under a nitrogen atmosphere and dissolved at −40°C. Volatile by-products and excess hydrogen fluoride were removed under a dynamic vacuum at −78°C overnight, leaving a colorless, amorphous glass-like residue.

Synthesis of **α**-Fluorohydroxyacetic Fluoride

First, the FEP reactor was dried chemically with F_2. Sulfur tetrafluoride (216 mg, 2.00 mmol) and anhydrous hydrogen fluoride (approximately 0.5 mL) were then successively condensed into the reactions vessel at −196°C and agitated at −40°C. After cooling down to −196°C, glyoxylic acid monohydrate (92.05 mg, 1.00 mmol) was added under a nitrogen atmosphere and dissolved at −40°C. Volatile by-products and excess hydrogen fluoride were removed under a dynamic vacuum at −78°C overnight, leaving a colorless, amorphous glass-like residue.

Synthesis of Protonated **α**-Fluorohydroxyacetic Acid

For the synthesis of protonated α-fluorohydroxyacetic acid, arsenic pentafluoride (510 mg, 3.00 mmol) (**3**) was condensed into a reactor (FEP tube), followed by excess anhydrous hydrogen fluoride (aHF) at −196°C. The mixture was warmed up to 0°C to form the superacidic medium. After cooling down to −196°C again, glyoxylic acid monohydrate (92 mg, 1.00 mmol) was added and subsequently dissolved at −45°C. After warming up to 0°C, the mixture was agitated again and dried overnight at −78°C under a dynamic vacuum, leaving a colorless powder.

Results and Discussion

NMR Spectroscopy

Glyoxylic Acid Monohydrate (GAM) in D_2O

^{1}H NMR (400 MHz, Deuterium Oxide) δ [ppm] = 5.35 (s, 1H).
^{13}C {^{1}H} NMR (101 MHz, Deuterium Oxide) δ [ppm] = 173.17, 86.14.

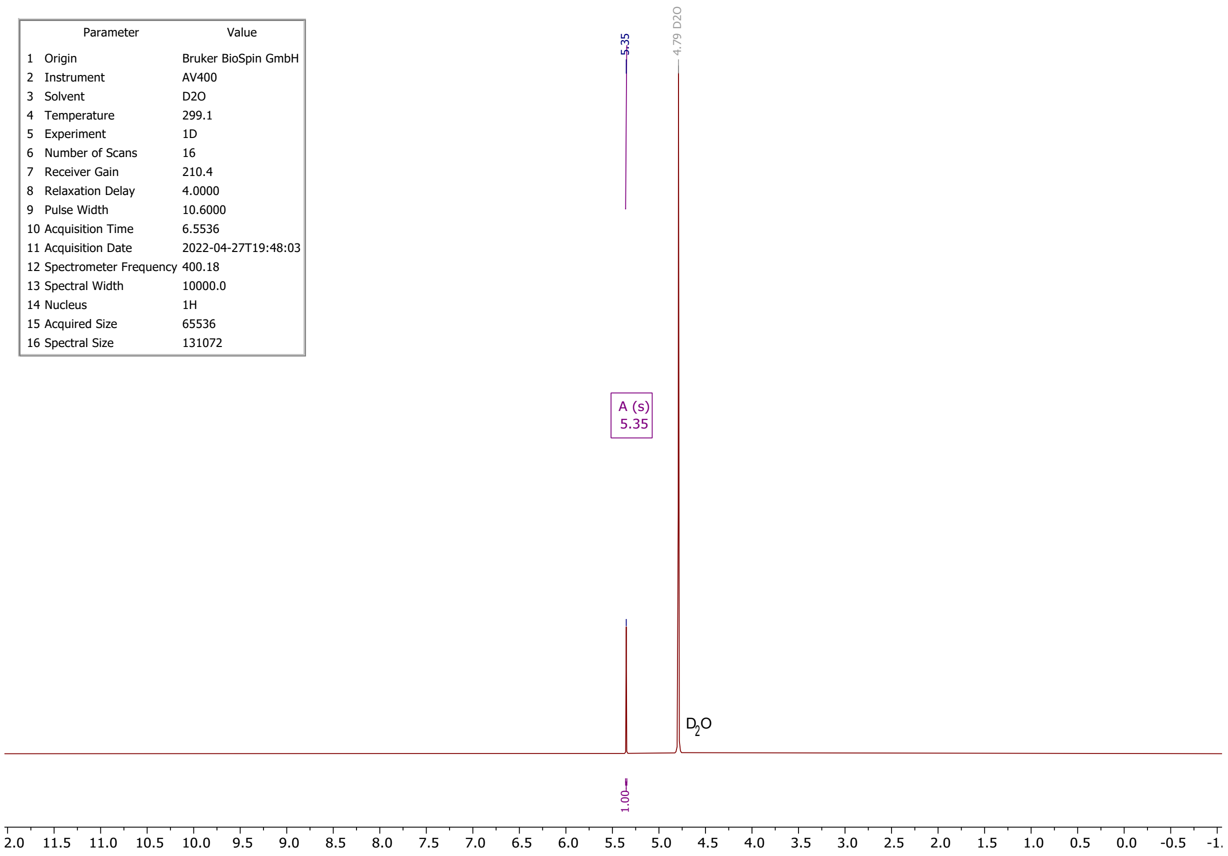

Figure S1. ^{1}H NMR spectrum of GAM in D_2O at room temperature.

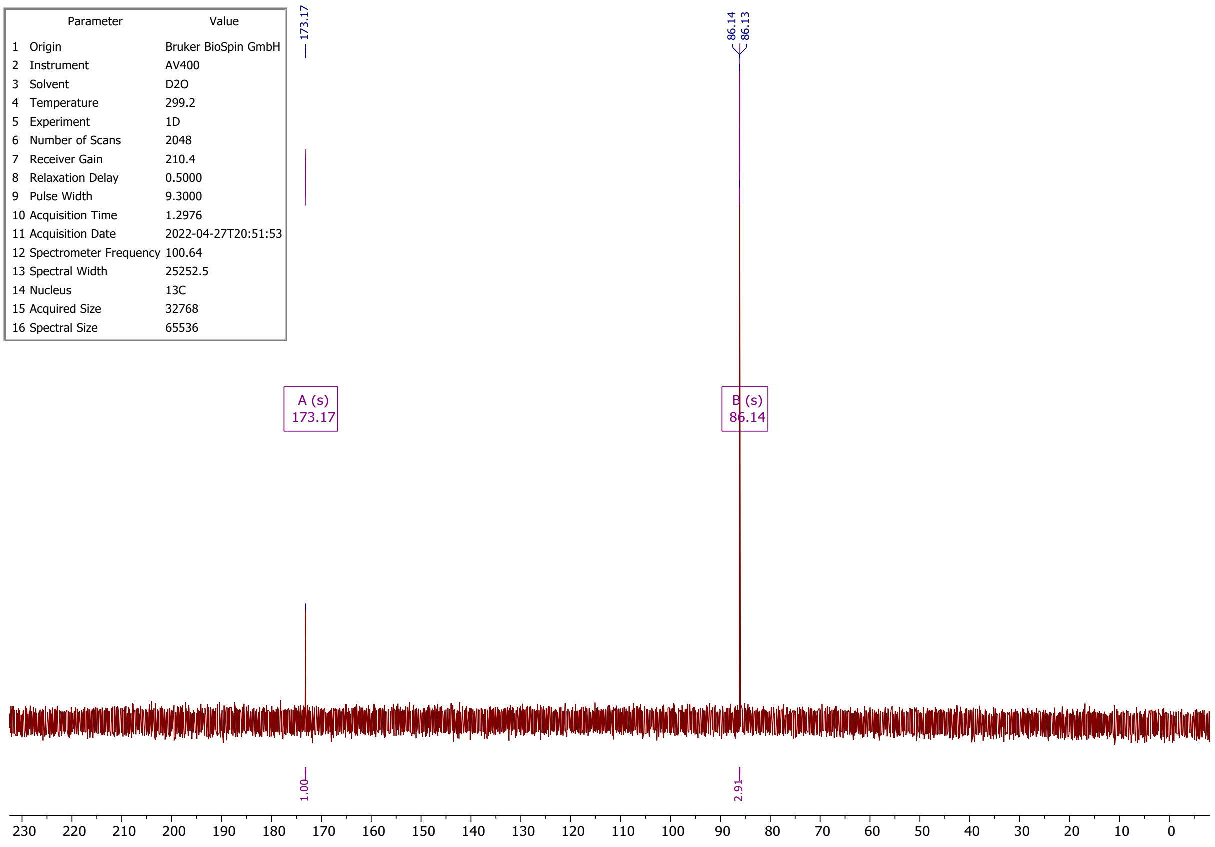

Figure S2. ^{13}C {^{1}H} NMR spectrum of GAM in D_2O at room temperature.

Glyoxylic Acid Monohydrate (GAM) dissolved in aHF

^{1}H NMR (400 MHz, aHF) δ [ppm] = 5.96 (d, *J* = 6.0 Hz), 5.87 (d, *J* = 8.1 Hz), 5.78 (s), 5.72 (s), 5.63 (d, *J* = 5.6 Hz), 5.57 (s), 5.50 (s).
^{19}F NMR (376 MHz, aHF) δ [ppm] = −129.63 (dd, *J* = 58.5, 22.3 Hz), −134.85 (d, *J* = 61.6 Hz).
^{13}C {^{1}H} NMR (101 MHz, aHF) δ [ppm] = 171.57 (d, *J* = 32.6 Hz), 169.11, 168.48, 168.32, 100.43 (d, *J* = 65.2 Hz), 99.11, 98.70, 98.07 (d, *J* = 64.8 Hz), 96.88.

Parameter		Value
1	Origin	JEOL
2	Solvent	aHF
3	Temperature	0.0
4	Experiment	1D
5	Receiver Gain	16.0
6	Relaxation Delay	4.0000
7	Pulse Width	5.7000
8	Acquisition Time	2.6214
9	Acquisition Date	2022-04-21T09:08:56
10	Spectrometer Frequency	399.78
11	Spectral Width	10000.0
12	Lowest Frequency	-1991.3
13	Nucleus	1H
14	Acquired Size	32768
15	Spectral Size	104858
16	Digital Resolution	0.10

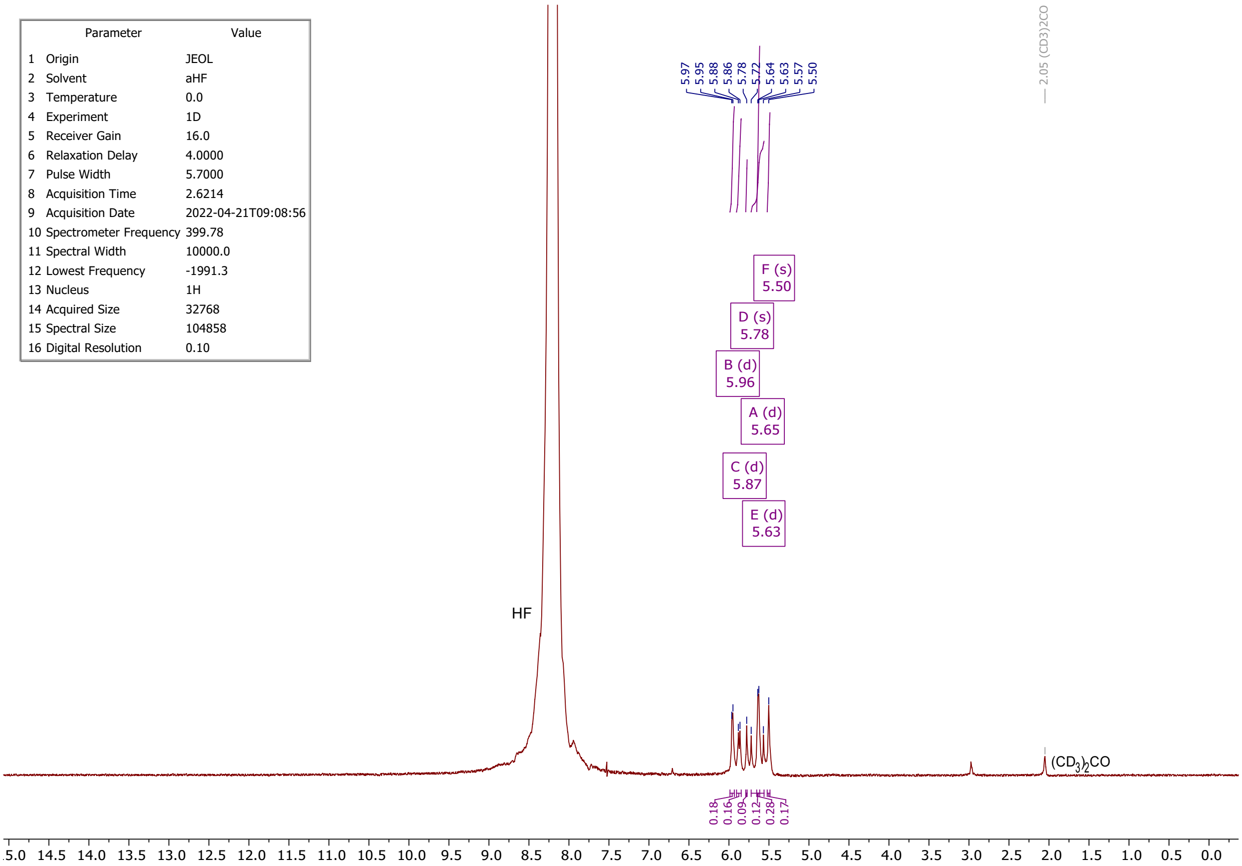

Figure S3. ^{1}H NMR spectrum of GAM in aHF at 0°C.

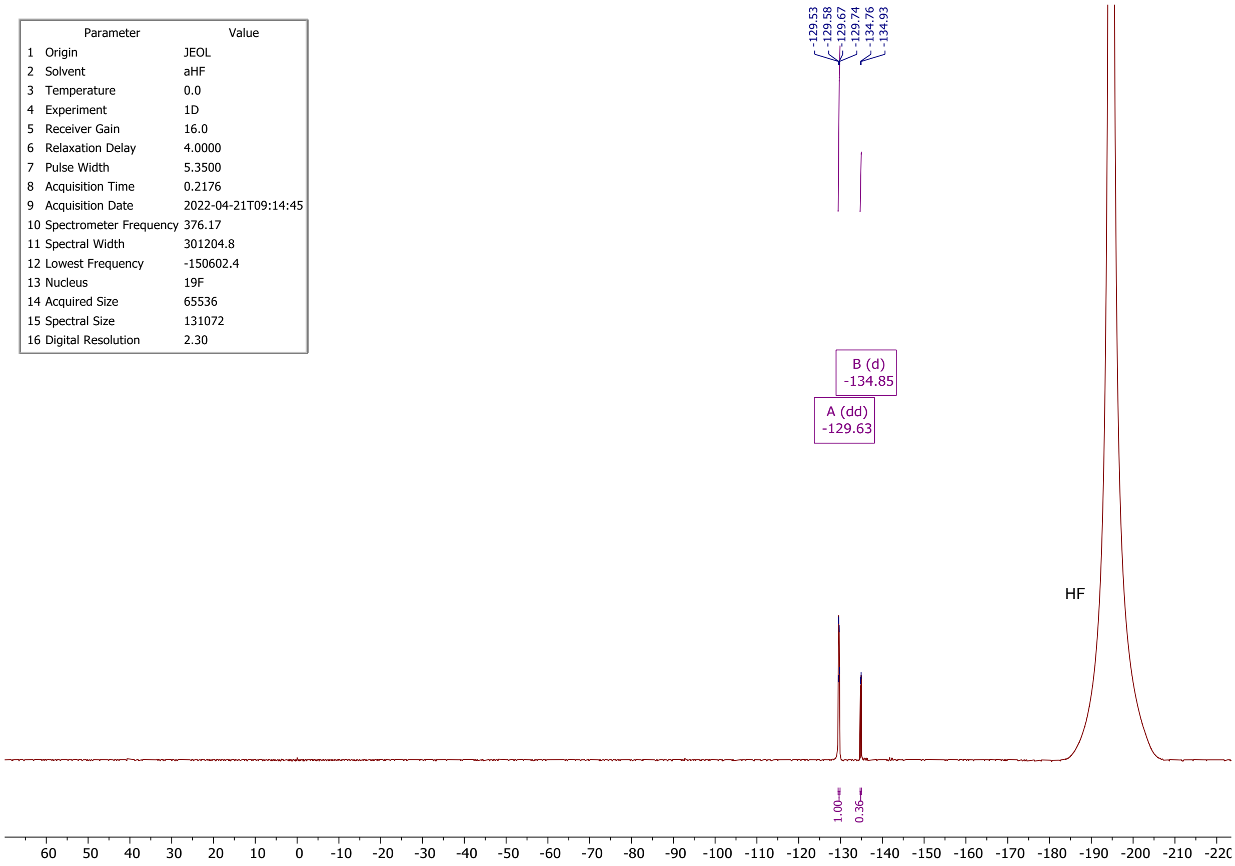

Figure S4. ^{19}F NMR spectrum of GAM in aHF at 0°C.

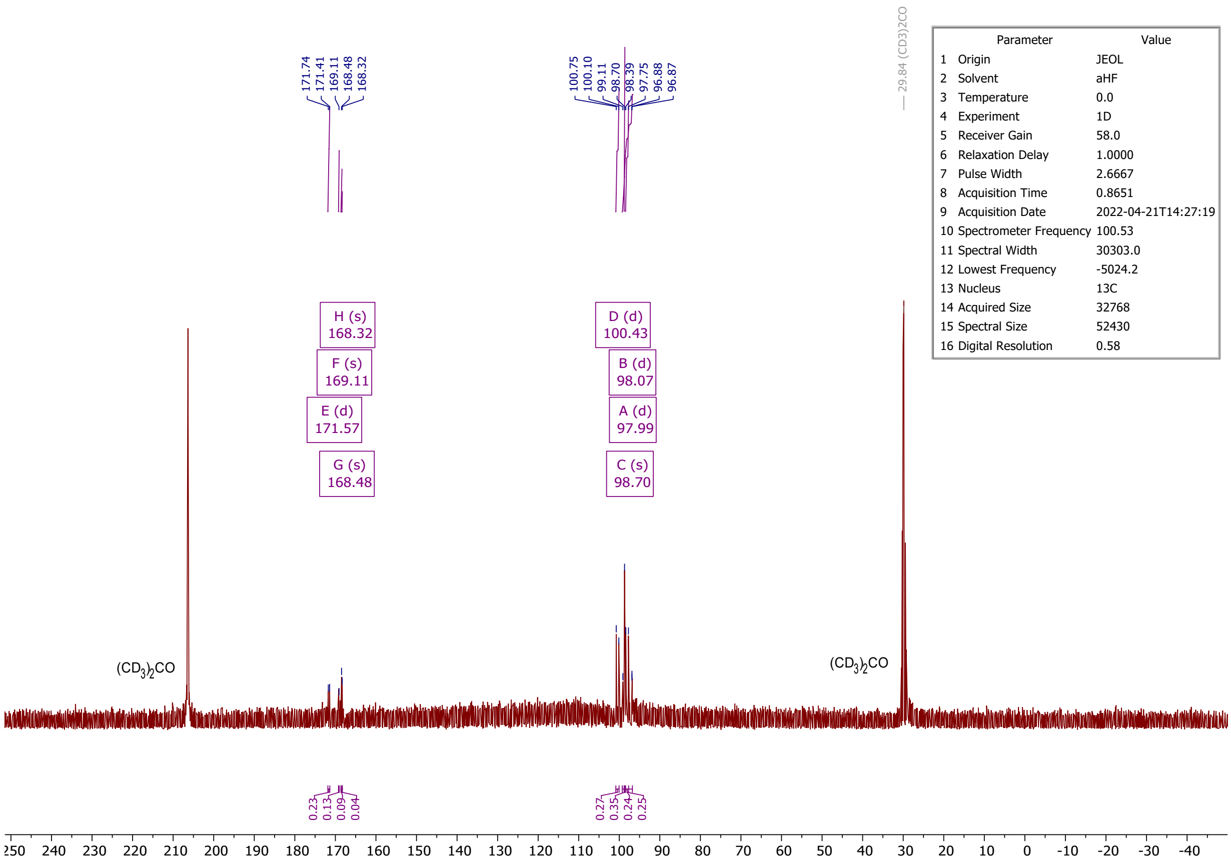

Figure S5. ^{13}C NMR spectrum of GAM in aHF at 0°C.

*2,2-Fluorohydroxyacetic Acid (**1**)*

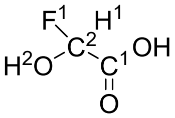

^{1}H NMR (400 MHz, aHF) δ [ppm] = 5.57 (d, *J* = 54.4 Hz, H1), 3.30 (s, br, H2).
^{19}F NMR (376 MHz, aHF) δ [ppm] = −130.38 (d, *J* = 54.2 Hz, F1).
^{13}C {^{1}H} NMR (101 MHz, aHF) δ [ppm] = 170.98 (d, *J* = 32.7 Hz, C1), 96.95 (d, *J* = 225.1 Hz, C2).

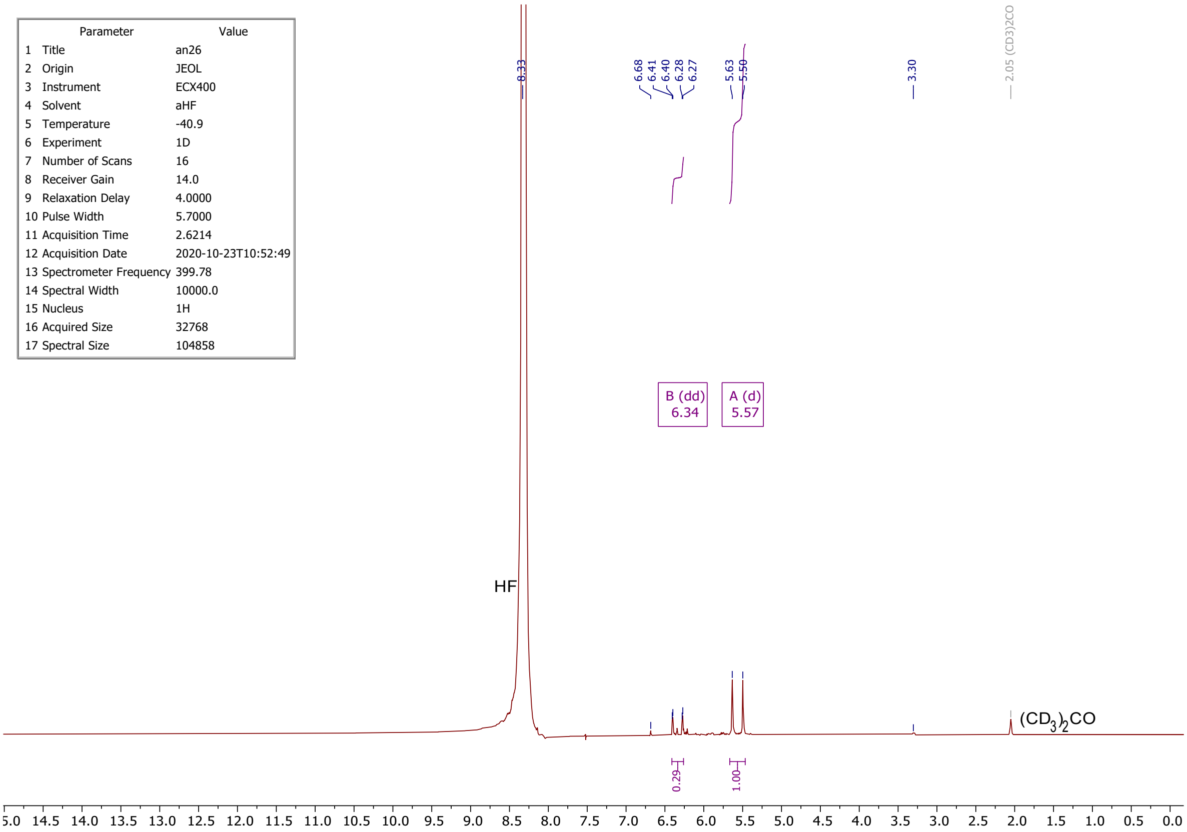

Figure S6. ^{1}H NMR spectrum of GAM with equimolar amounts of SF_4 in aHF at −40°C.

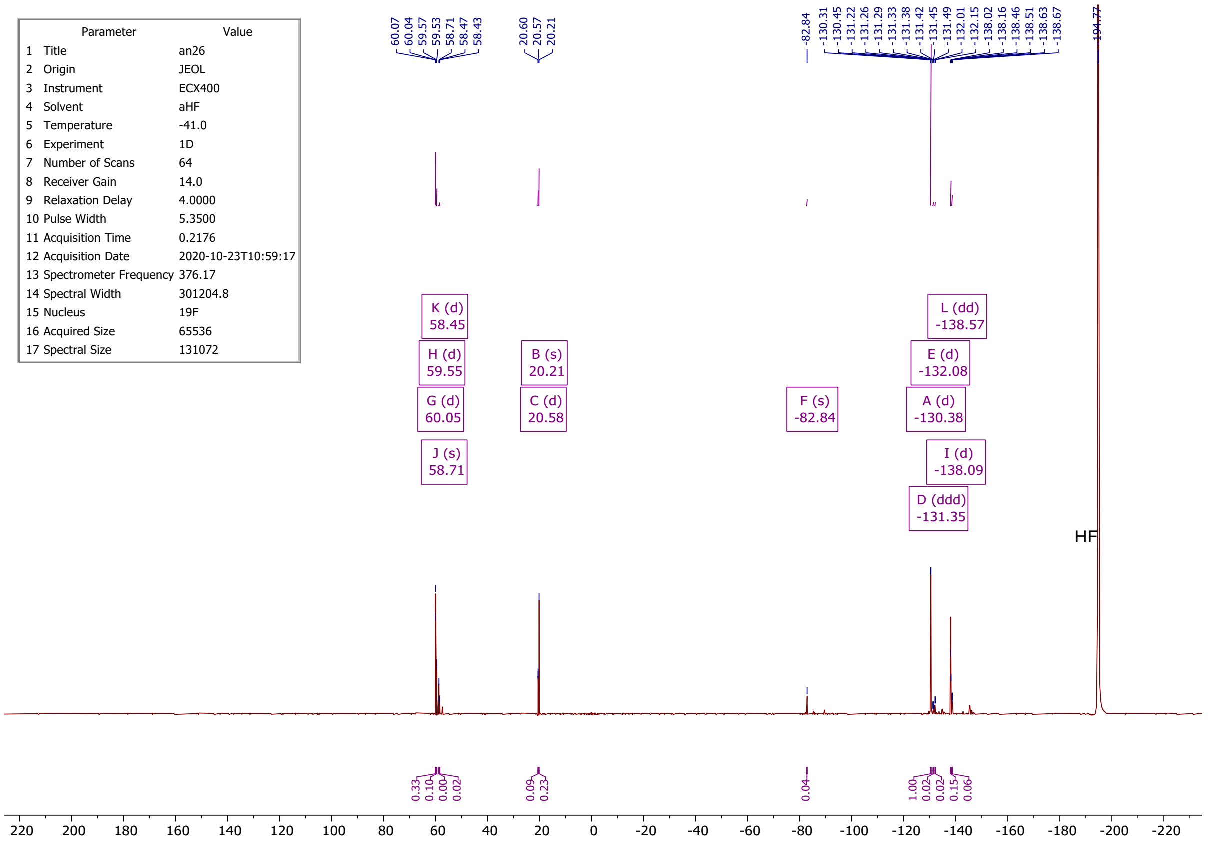

Figure S7. ^{19}F NMR spectrum of GAM with equimolar amounts of SF_4 in aHF at −40°C.

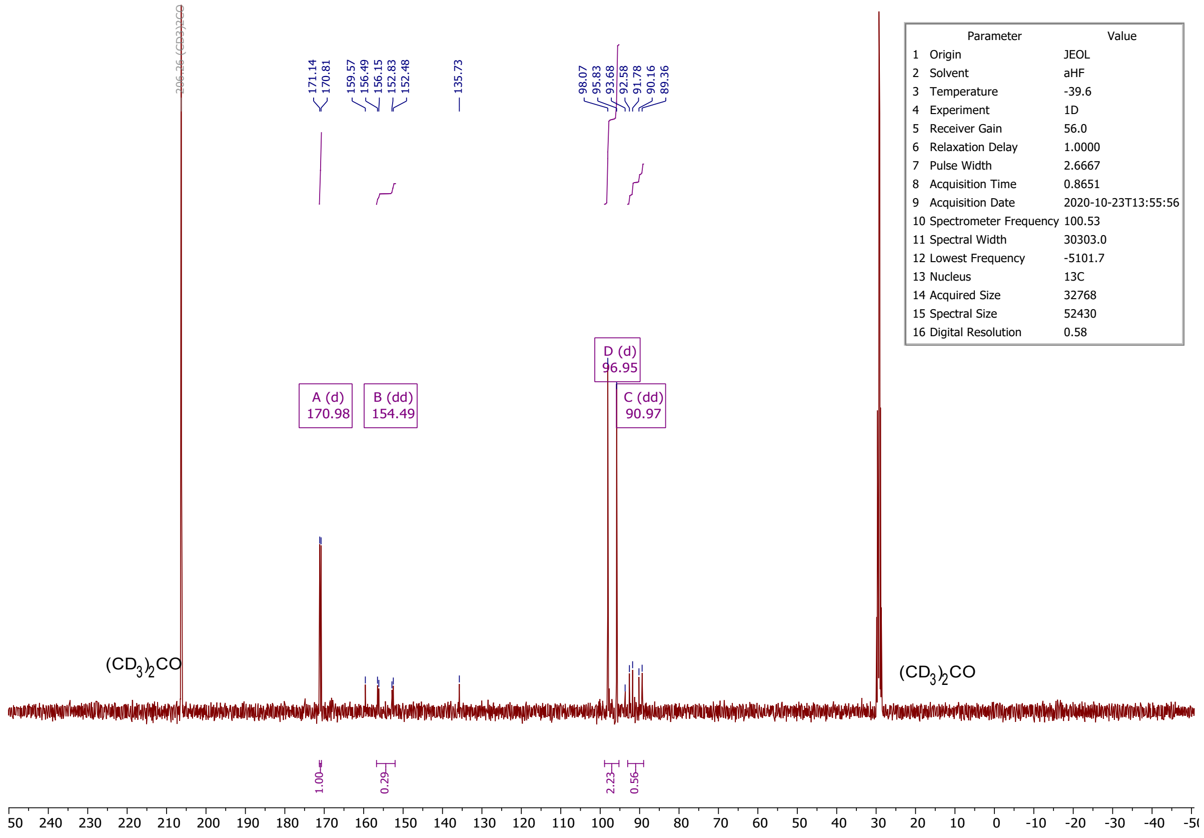

Figure S8. ^{13}C {^{1}H} NMR spectrum of GAM with equimolar amounts of SF_4 in aHF at −40°C.

*2,2-Fluorohydroxyacetyl Fluoride (**2**)*

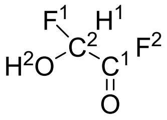

^{1}H NMR (400 MHz, SO_2) δ [ppm] = 7.00 (d, *J* = 51.2 Hz, H1).
^{19}F NMR (376 MHz, SO_2) δ [ppm] = 23.63 (d, *J* = 16.3 Hz), 22.97 (d, *J* = 14.1 Hz, F2), −134.67 (d, *J* = 53.7 Hz F1).
^{13}C {^{1}H} NMR (101 MHz, SO_2) δ [ppm] = 154.33 (dd, *J* = 368.7 Hz, 34.8 Hz, C1), 92.59 (dd, *J* = 241.3 Hz, 82.4 Hz, C2).

2 is generated by dissolving a twofold amount of SF_4 (with respect to GAM) in aHF and successively adding GAM at −40°C. After removing all volatile products like SOF_2 and the solvent overnight, the residue was redissolved in aHF. ^{1}H, ^{19}F, and ^{13}C NMR spectra (Figure S9, Figure S10, and Figure S11) were measured at −40°C. The ^{19}F NMR spectrum indicates an HF addition to **2**, illustrated in Equation S1.

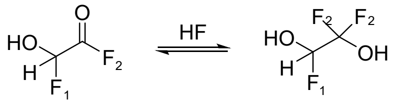

(S1)

2 **4**

The signal at −138.79 ppm with a dt-splitting pattern ($^2J_{HF}$ = 53.7 Hz, $^3J_{FF}$ = 9.35 Hz) is assigned to the F1 atom of the HF-adduct (**4**). The triplet coupling was calculated manually. The ^{19}F signal of the F2 atom occurs at −83.46 ppm and is consistent with reported perfluorinated alcohols.[12] The respective proton signal is observed at 6.28 ppm with a dd-splitting pattern ($^2J_{HF}$ = 51.0 Hz, $^3J_{HF}$ = 3.7 Hz). The ^{13}C resonance of the CHF(OH) group is noticed at 91.31 ppm (dd, $^1J_{CF}$ = 242.9 Hz, $^2J_{CF}$ = 80.2 Hz). The equilibrium displayed in Equation S1 is shifted to **2** by removing the solvent, successively warming the residue up to 0°C, and trapping the gas phase into a second vessel at −196°C. The condensate was dissolved in SO_2, and NMR spectra (Figure S12, Figure S13, and Figure S14) show **2** as the only organic compound.

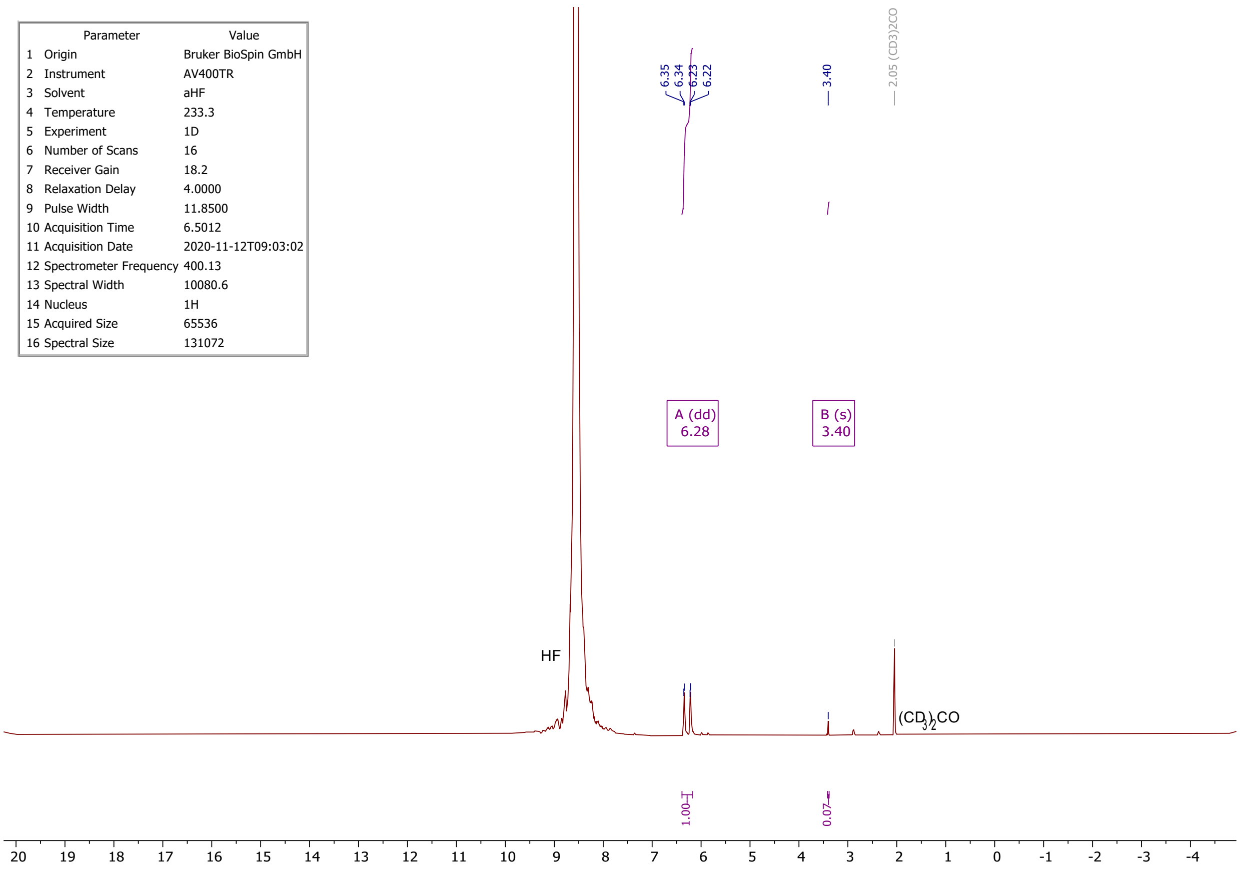

Figure S9. ^{1}H NMR spectrum of GAM with a twofold amount of SF_4 in aHF at −40°C.

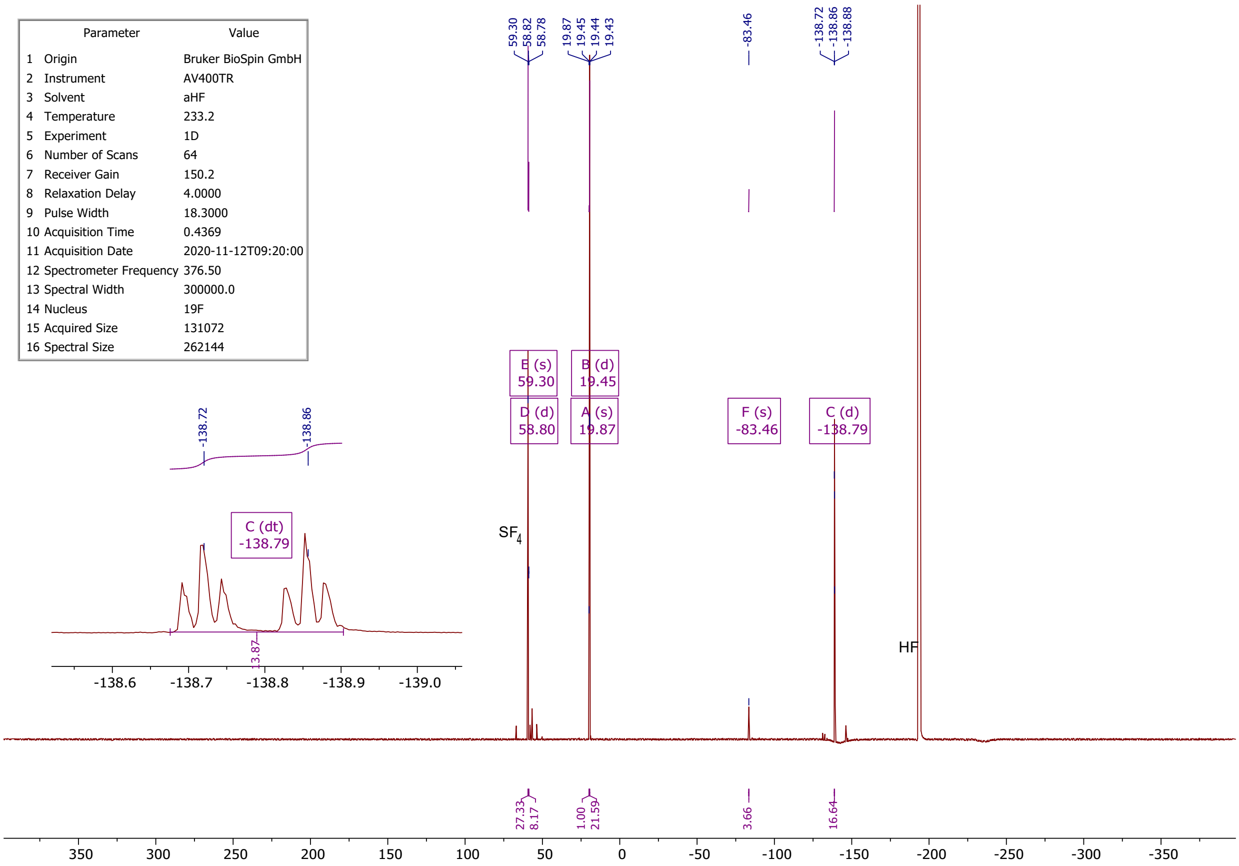

Figure S10. ^{19}F NMR spectrum of GAM with a twofold amount of SF_4 in aHF at −40°C.

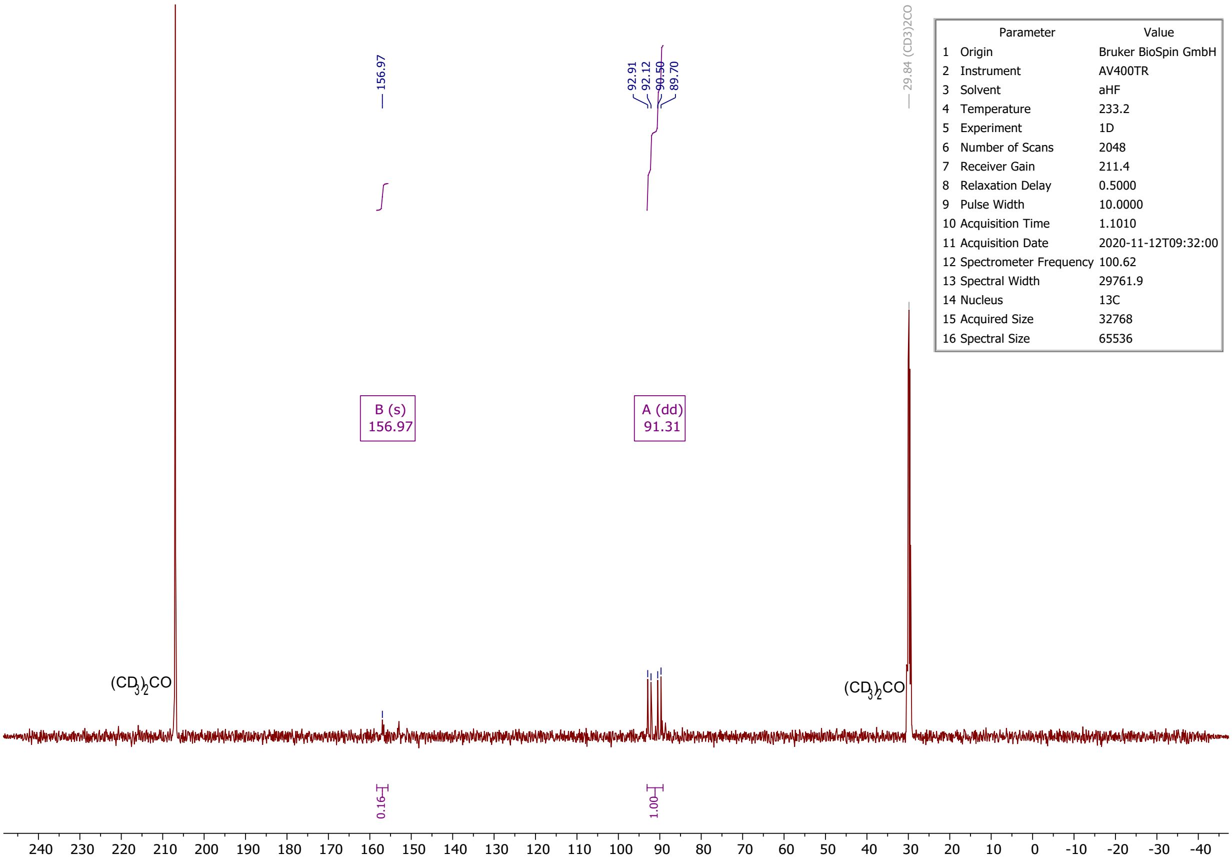

Figure S11. ^{13}C {^{1}H} NMR spectrum of GAM with a twofold amount of SF_4 in aHF at −40°C.

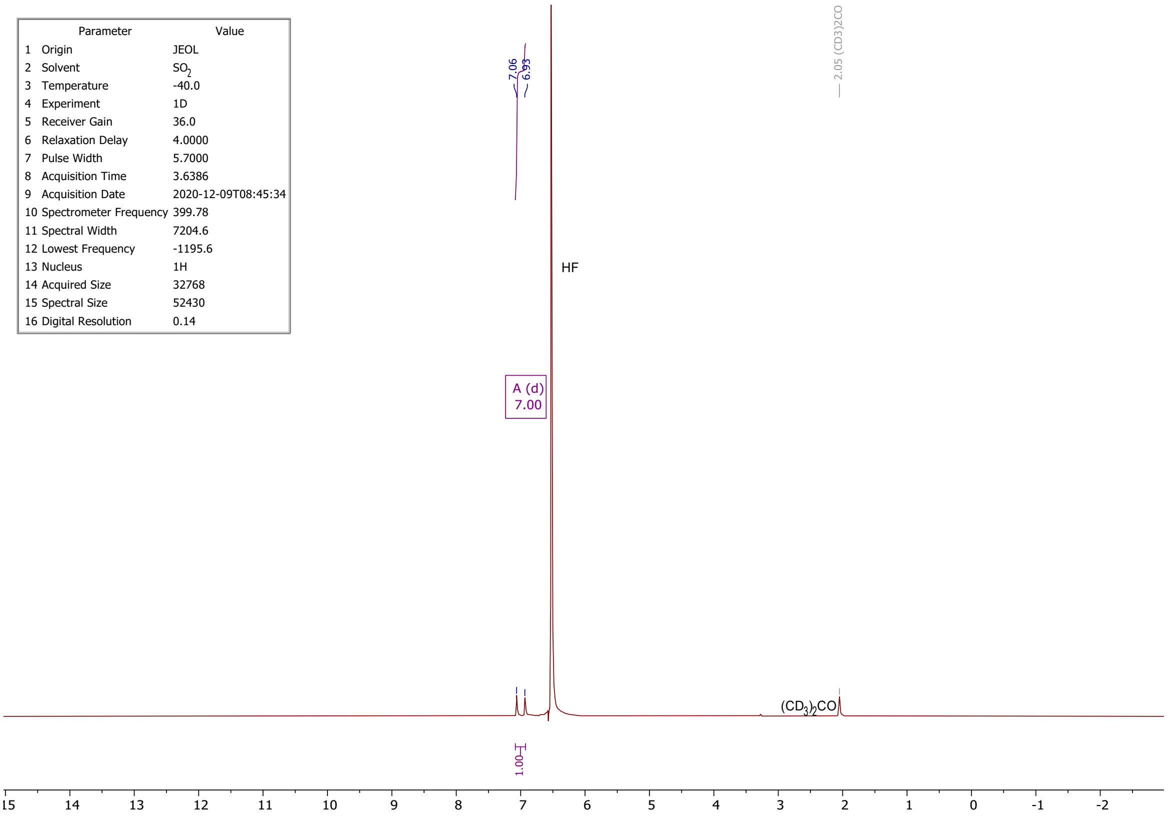

Figure S12. ^{1}H NMR spectrum of **2** in SO_2 at −40°C.

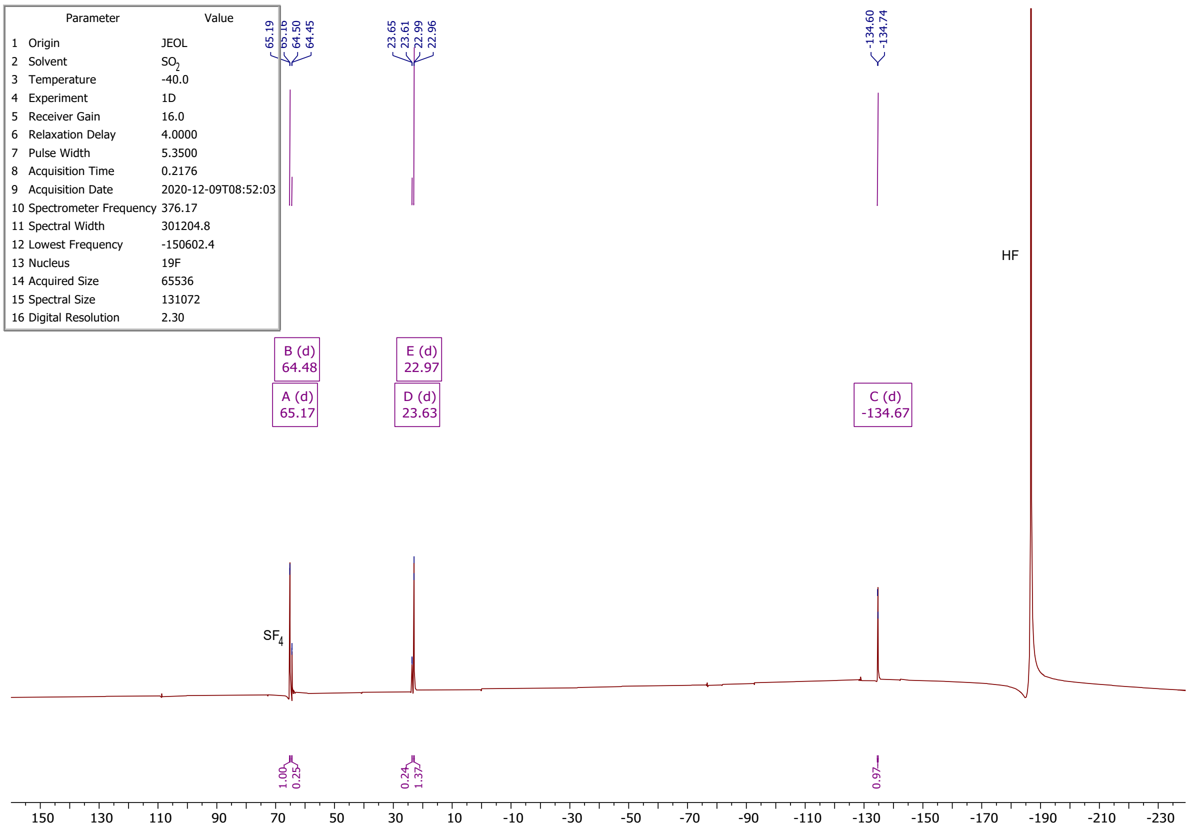

Figure S13. ^{19}F NMR spectrum of **2** in SO_2 at −40°C.

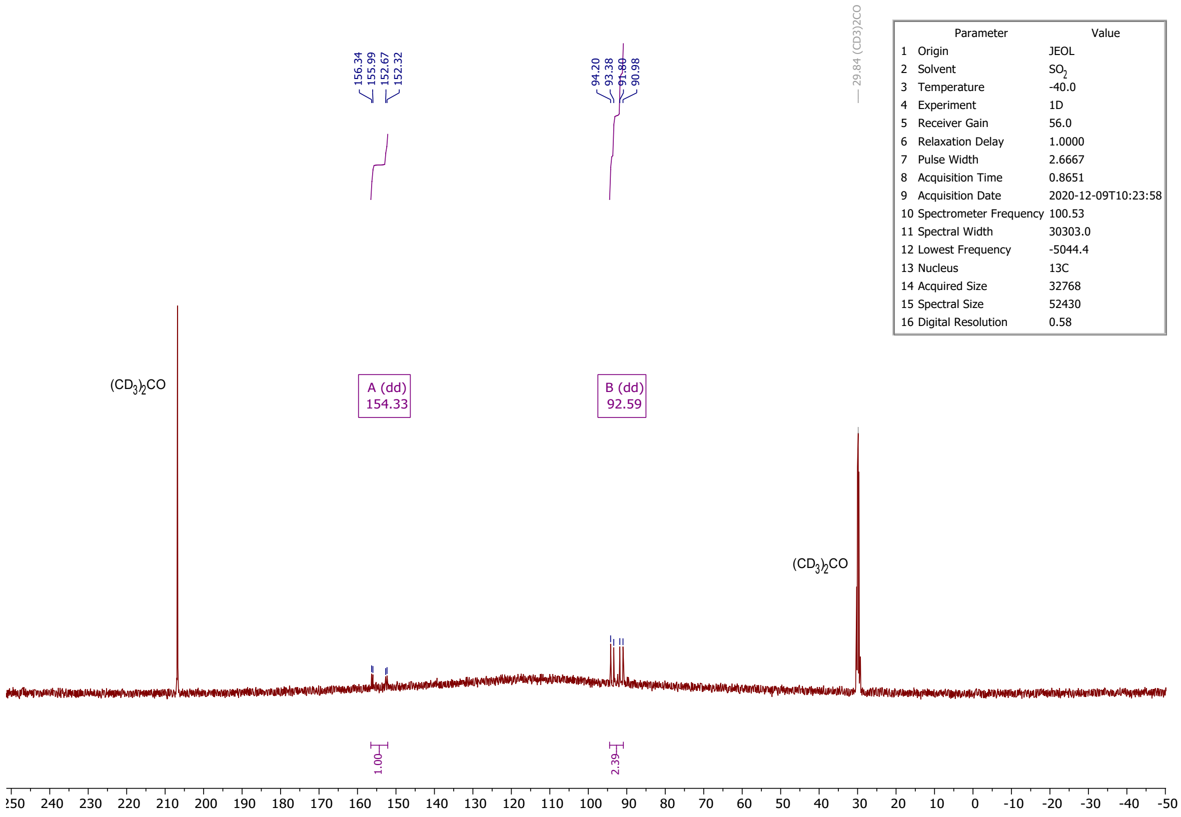

Figure S14. ^{13}C {^{1}H} NMR spectrum of **2** in SO_2 at −40°C.

*Protonated α-Fluorohydroxyacetic Acid [FHA-1H][AsF$_6$] (**3**)*

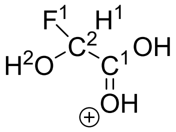

^{1}H NMR (400 MHz, aHF): δ [ppm] = 9.48 (s, H_3O^+) 5.61 (d, *J* = 54.9 Hz; $^{2}J_{HF}$, H1), 3.39 (s, H2).
^{19}F NMR (376 MHz, aHF): δ [ppm] = −128.88 (d, *J* = 53.9 Hz; $^{2}J_{HF}$, F1).
^{13}C {^{1}H} NMR (101 MHz, aHF): δ [ppm] = 184.90 (d, *J* = 34.0 Hz; $^{2}J_{CF}$, C1), 97.00 (d, *J* = 227.1 Hz; $^{1}J_{CF}$, C2).

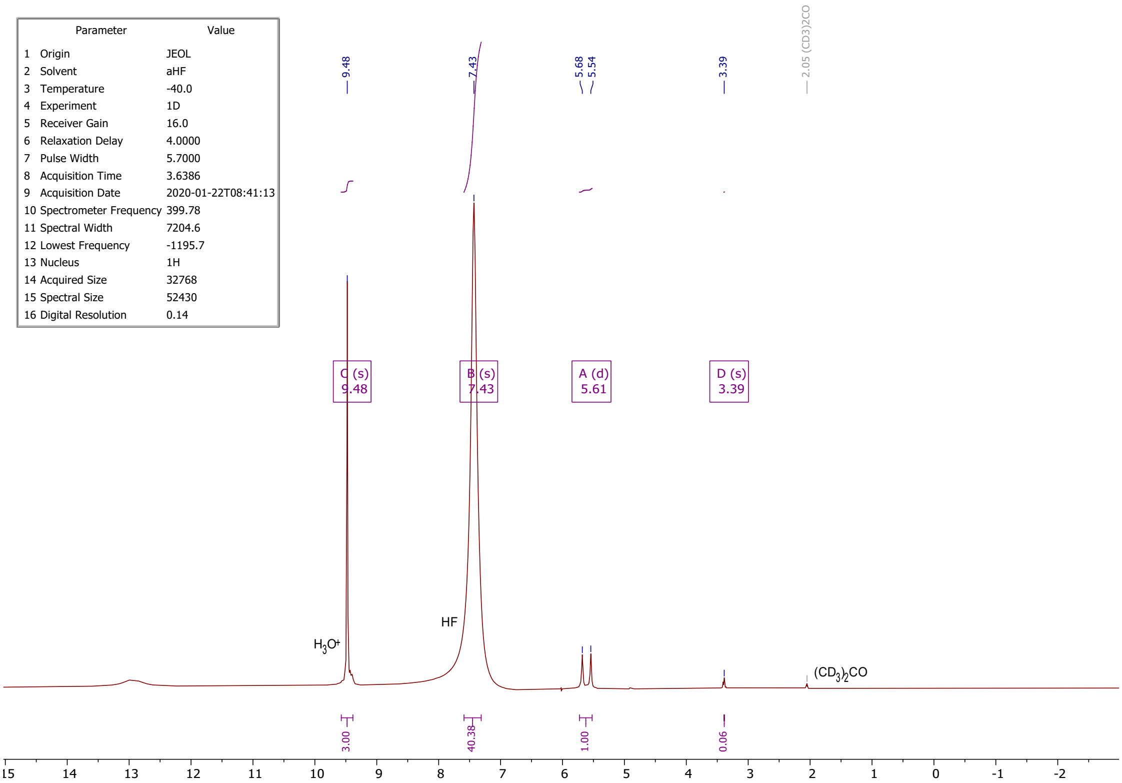

Figure S15. ^{1}H NMR spectrum of **3** in aHF at −40°C.

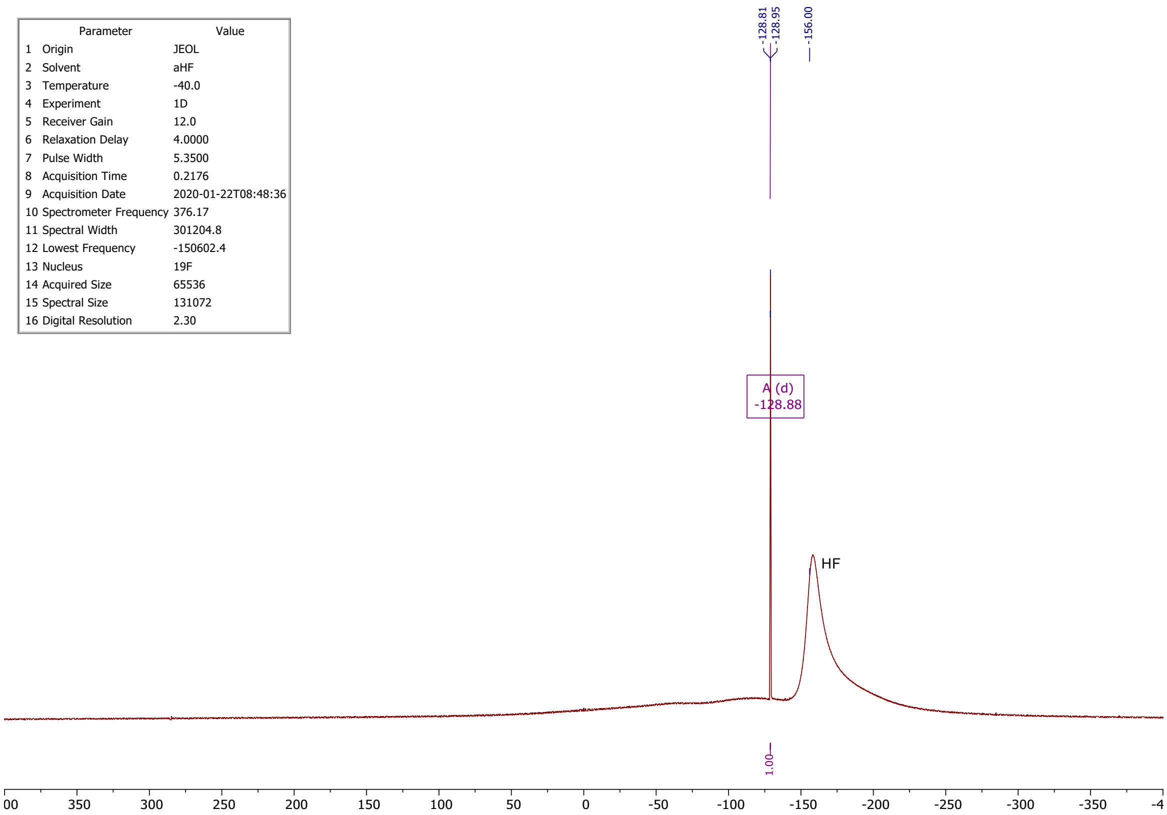

Figure S16. ^{19}F NMR spectrum of **3** in aHF at −40°C.

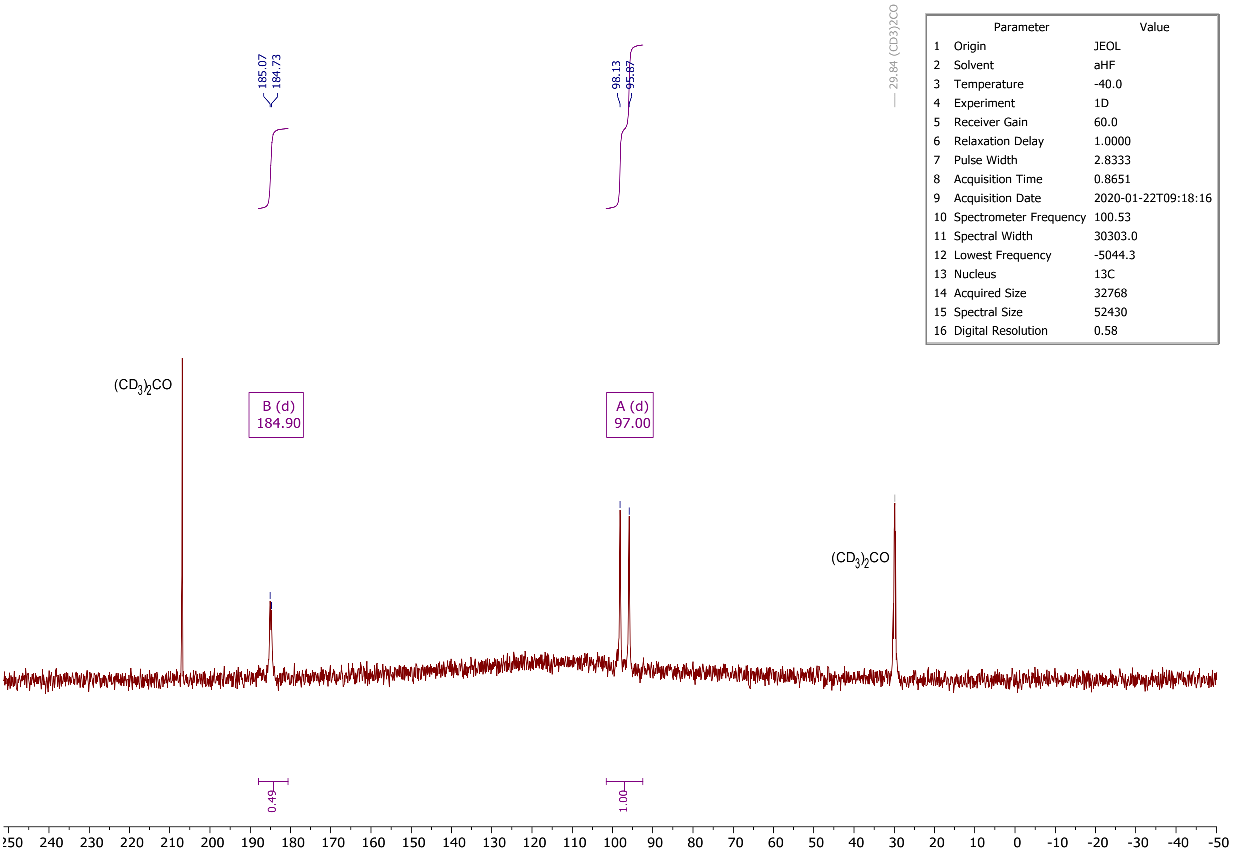

Figure S17. $^{13}C\{^1H\}$ NMR spectrum of **3** in aHF at −40°C.

Vibrational Spectroscopy

Table S1. Experimental vibrational frequencies [cm^{-1}] of [FHA-1H][AsF_6] (**3**) and calculated frequencies of $[FHA\text{-}1H]^+$ (B3LYP/aug-cc-pVTZ level of theory).

**[FHA-1H][AsF_6]**		**$[FHA\text{-}1H]^+$**	
**IR (exp.)[a]**	**Raman (exp.)[a]**	**$\tilde{\nu}$ (IR/Ra) (calc.)[b],[c]**	**Assignment**
		3769 (212/82)	ν(OH)
		3615 (256/84)	ν(OH)
		3484 (336/31)	ν(OH)
	3127 (6)		
	3013 (11)	3067 (5/70)	ν(CH)
1705 (m)		1685 (268/1)	ν_{as}(CO)
	1623 (4)		
1541 (m)	1567 (7)	1567 (169/4)	ν_s(CO)
1412 (m)	1407 (2)	1422 (74/1)	ω(CH)
1362 (m)	1335 (7)	1354 (10/3)	δ(CCH)
1225 (m)	1247 (3)	1241 (198/6)	δ(COH)
	1206 (2)	1193 (33/4)	δ(COH)
1165 (m)	1185 (10)	1189 (175/2)	δ(FCH)
1151 (m)	1162 (3)	1154 (102/1)	ν(CO)
1022 (m)	1027 (4)	1066 (146/5)	ν(CF)
1007 (m)			
897 (m)	903 (8)	883 (27/4)	ν(CC)
854 (m)			
814 (m)		798 (25/0)	γ(OH)
764 (m)	747 (14)	760 (123/4)	δ(CCO_2)
696 (s)		693 (116/2)	δ(CCO)
		608 (5/1)	δ(CCO)
552 (m)		549 (49/1)	δ(COC)
	469 (13)	440 (0/1)	δ(CCO)
		324 (117/1)	γ(OH)
		303 (61/0)	δ(CCO)
		238 (1/1)	δ(CCF)
		58 (3/1)	τ(CC)
	711 (97)		$[AsF_6]^-$
675 (m)	676 (100)		$[AsF_6]^-$
	566 (31)		$[AsF_6]^-$
	369 (68)		$[AsF_6]^-$

[a] Abbreviations for IR intensities: vs = very strong, s = strong, m = medium, w = weak. Experimental Raman intensities are relative to a scale of 1 to 100. [b] Calculated on the B3LYP/aug-cc-pVTZ level of theory. [c] IR intensities in km/mol; Raman intensities in Å^4/u.

Table S2. Experimental and calculated IR frequencies [cm^{-1}] of FHA-F (**2**). Calculated on the B3LYP/aug-cc-pVTZ level of theory.

FHA-F (exp.)[a]	FHA-F (calc.)[b],[c]	Assignment
	3800 (79)	ν(OH)
	3080 (19)	ν(CH)
1894 (m)	1907 (246)	ν(C=O)
	1453 (15)	δ(CH)
1373 (m)	1362 (10)	ω(CH)
1360 (m)		
1348 (m)		
1250 (m)	1278 (73)	δ(CCH)
1173 (w)	1189 (257)	ν(CF)
1128 (m)	1131 (84)	ν(C–O)
1051 (m)		
1016 (m)	1012 (177)	ν(CF)
822 (m)	832 (53)	ν(CC)
744 (m)	765 (42)	γ(COF)
689 (m)	693 (46)	δ(COF)
590 (w)		
581 (w)	579 (23)	δ(COF)
571 (w)		
517 (w)		
498 (m)		
474 (w)		
438 (m)		
420 (m)	418 (16)	δ(CCO)
	321 (98)	ω(OH)
	255 (3)	δ(CCF)
	235 (3)	δ(CCF)
	48 (4)	τ(CC)
1286 (m)		SF_4
885 (w)		SF_4
708 (m)		SF_4
623 (m)		SF_4
609 (vs)		SF_4
552 (m)		SF_4
534 (m)		SF_4

[a] Abbreviations for IR intensities: vs = very strong, s = strong, m = medium, w = weak. [b] Calculated on the B3LYP/aug-cc-pVTZ level of theory. [c] IR intensities in km/mol.

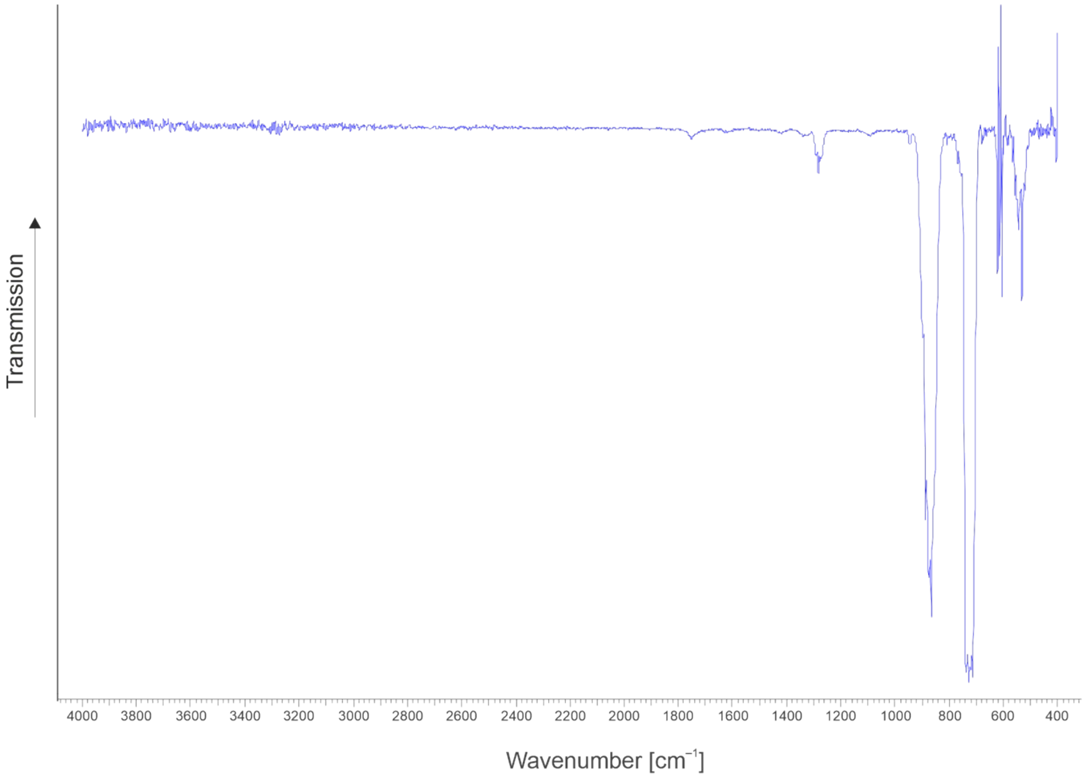

Figure S18. Reference spectrum of gaseous SF_4.

$\tilde{\nu}$ [cm^{-1}]= 1284 (m), 884 (w), 729 (vw), 622 (m), 612 (m), 603 (m), 555 (m), 544 (m), 532 (m), 406 (m), 397 (m).

Table S3. Quantum chemically calculated vibrational frequencies of **1**. Calculated on the B3LYP/aug-cc-pVTZ level of theory.

**FHA**	
$\tilde{\nu}$ (IR/Ra)	Assignment
3799 (67/124)	ν(OH)
3732 (78/104)	ν(OH)
3080 (21/71)	ν(CH)
1823 (302/12)	ν(C=O)
1461 (20/2)	δ(CCH)
1381 (47/3)	δ(COH)
1360 (19/3)	ω(CH)
1250 (52/4)	δ(COH)
1170 (261/2)	ν(C–O)
1138 (88/4)	ν(C–O)
1011 (191/3)	ν(CF)
864 (23/10)	ν(CC)
789 (70/0)	γ(CO)
660 (65/3)	δ(CCO)
591 (74/1)	ω(OH)
571 (28/2)	δ(CCO)
419 (17/1)	δ(COF)
326 (94/2)	ω(OH)
267 (11/1)	δ(CCO)
240 (3/1)	δ(CCF)
40 (5/1)	τ(CC)

Single-Crystal X-ray Structure Analysis

Crystal Structure of [FHA-1H][AsF$_6$]

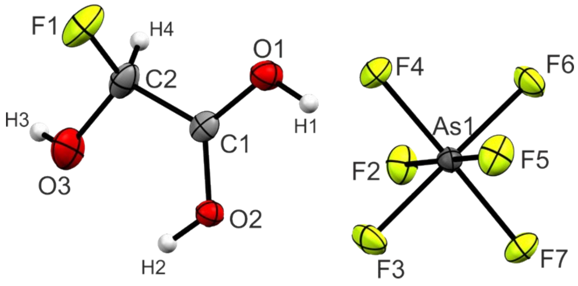

Figure S19. Projection of the asymmetric unit of **3**. Thermal ellipsoid displacement probability set at 50%, hydrogen atoms displayed as spheres of arbitrary radius.

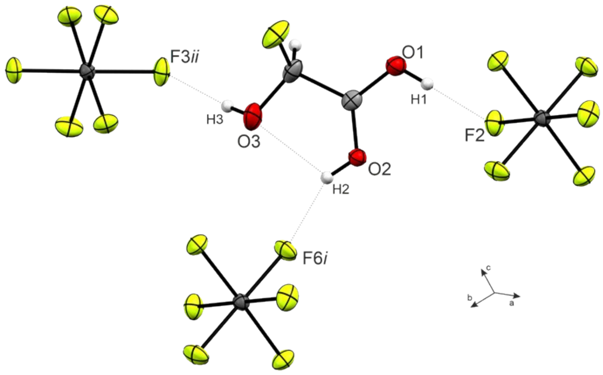

Figure S20. Hydrogen bonds of the cation in the crystal packing of **3**. Thermal ellipsoid displacement probability set at 50%, hydrogen atoms displayed as spheres of arbitrary radius.

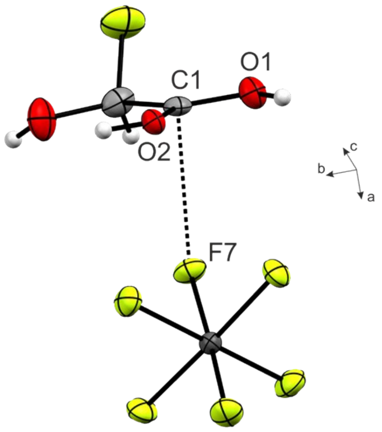

Figure S21. Non-hydrogen bonded cation-anion interaction in the crystal packing of **3**. Thermal ellipsoid displacement probability set at 50%, hydrogen atoms displayed as spheres of arbitrary radius.

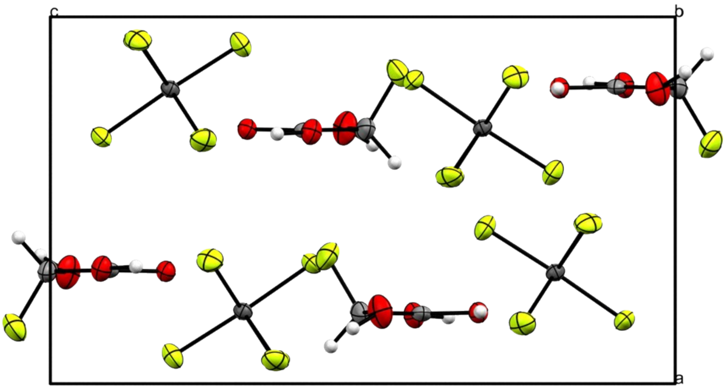

Figure S22. Crystal packing of **3** with a view along the *b*-axis. Thermal ellipsoid displacement probability set at 50%, hydrogen atoms displayed as spheres of arbitrary radius.

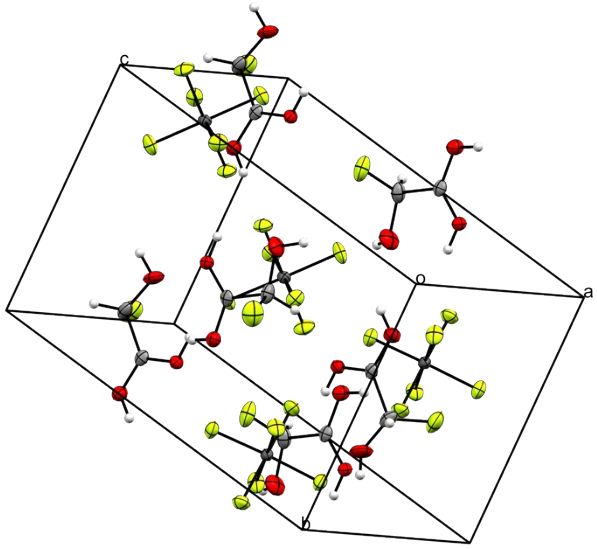

Figure S23. Crystal packing of **3**. Thermal ellipsoid displacement probability set at 50%, hydrogen atoms displayed as spheres of arbitrary radius.

Table S4. Bond lengths [Å], bond angles, and torsion angles [°] of **3**.

**Bond lengths [Å]**			
C1–C2	1.515(5)	As1–F2	1.754(2)
C1–O1	1.258(4)	As1–F3	1.727(2)
C1–O2	1.272(4)	As1–F4	1.703(2)
C2–O3	1.355(5)	As1–F5	1.698(3)
C2–F1	1.376(5)	As1–F6	1.727(2)
		As1–F7	1.716(2)
**Bond angles [°]**			
O1–C1–C2	117.4(3)	F3–As1–F2	89.03(13)
O2–C1–C2	122.0(3)	F4–As1–F6	90.47(12)
O1–C1–O2	120.6(3)	F5–As1–F7	91.96(12)
O3–C2–C1	106.6(3)		
F1–C2–C1	106.1(4)		
O3–C2–F1	111.9(4)		
**Dihedral angles [°]**			
O3–C2–C1–O1	179.5(4)		
F1–C2–C1–O1	−61.2(5)		
O3–C2–C1–O2	−1.0(6)		
F1–C2–C1–O2	118.3(4)		
**Intermolecular interactions D(−H)···A [Å]**			
O1(−H1)···F2	2.579(4)		
O2(−H2)···O3	2.587(4)		
O2(−H2)···F6*i*	2.669(3)		
O3(−H3)···F3*ii*	2.826(4)		
C1···F7	2.733(5)		

Table S5. Summary of the X-ray diffraction data collection and refinement.

	**[$C_2H_4FO_3$][AsF_6]**
Formula	$C_2H_4F_7O_3As$
M_r [g mol^{-1}]	283.97
Crystal size [mm^3]	0.80 × 0.170 × 0.150
Crystal system	orthorhombisch
Space group	$P2_12_12_1$
a [Å]	7.4858(3)
b [Å]	8.0027(3)
c [Å]	12.7743(5)
α [deg]	90
β [deg]	90
γ [deg]	90
V [Å^3]	765.27(5)
Z	4
ρ_{calc} [g cm^{-3}]	2.465
μ [mm^{-1}]	4.549
λ(Mo-K_α) [Å]	0.71073
F(000)	544
T [K]	121(2)
h,k,l range	–10:10, –11:11, –18:18
Refl. measured	2544
Refl. unique	2257
R_{int}	0.0437
Parameters	135
R(*F*)/*w*R(F^2) [a] (all reflexions)	0.0416/0.0631
Weighting scheme	calc
S (GooF)[d]	1.028
Residual density [e Å^{-3}]	0.797/–0.544
Device type	Oxford XCalibur
Solution	SHELXT[4]
Refinement	SHELXL-2018/1[5]
CCDC	2173682

Theoretical Study

α-Fluorohydroxyacetic Acid (FHA)

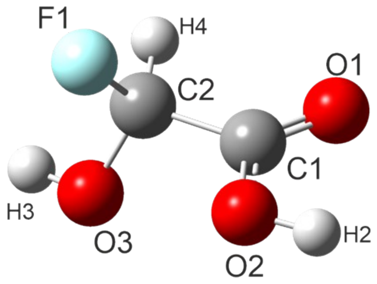

Figure S24. Optimization of the gas-phase structure of FHA. Calculated on the B3LYP/aug-cc-pVTZ level of theory.

Table S6: Standard orientation of FHA. Calculated on the B3LYP/aug-cc-pVTZ level of theory.

Atomic Type	X	Y	Z
F	−1.244502	0.840437	−0.813111
O	1.067135	0.952424	0.707265
O	1.637915	−0.829520	−0.548746
O	−1.270126	−0.612020	0.939554
C	−0.695407	−0.320106	−0.276339
C	0.807254	−0.108723	−0.065397
H	−2.144245	−0.993753	0.803175
H	−0.835056	−1.104689	−1.020467
H	2.029356	1.020418	0.801123

α-Fluorohydroxyacetic Acid (FHA-F)

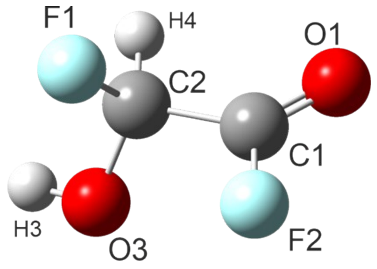

Figure S25. Optimization of the gas-phase structure of FHA-F. Calculated on the B3LYP/aug-cc-pVTZ level of theory.

Table S7. Standard orientation of FHA-F. Calculated on the B3LYP/aug-cc-pVTZ level of theory.

Atomic Type	X	Y	Z
F	−1.207018	0.951366	−0.689844
O	1.704702	−0.629905	−0.646942
O	−1.255582	−0.777422	0.790538
C	−0.671096	−0.284040	−0.349645
C	0.820887	−0.094466	−0.076235
H	−2.121126	−1.147330	0.583809
H	−0.791198	−0.930881	−1.219306
F	1.031532	0.782841	0.916734

Protonated α-Fluorohydroxyacetic Acid ([FHA-1H]+)

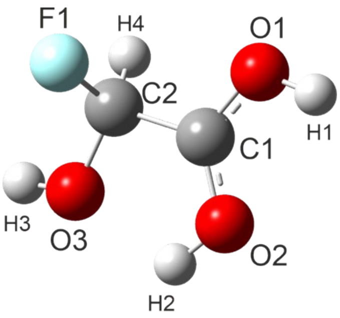

Figure S26. Optimization of the gas-phase structure of [FHA-1H]+. Calculated on the B3LYP/aug-cc-pVTZ level of theory.

Table S8. Standard orientation of [FHA-1H]+. Calculated on the B3LYP/aug-cc-pVTZ level of theory.

Atomic Type	X	Y	Z
F	−1.093802	−1.203964	−0.538907
O	1.072924	1.210624	−0.363999
O	1.592691	−0.841310	0.270649
O	−1.386180	0.937700	0.174918
C	−0.739742	−0.251522	0.375409
C	0.734321	0.072000	0.080148
H	0.281933	1.795160	−0.442466
H	−2.296761	0.932295	0.500484
H	2.509407	−0.597144	0.029094
H	−0.853324	−0.673612	1.377163

References

1 L. Bayersdorfer, R. Minkwitz and J. Jander, Eine Infrarot-Tieftemperaturkvette fr die Vermessung temperaturempfindlicher Gase, Flssigkeiten und Feststoffe, *Z. Anorg. Allg. Chem.*, 1972, **392**, 137–142.
2 CrysAlisCCD, *Version 1.171.35.11 (release 16-05-2011 CrysAlis 171.NET)*, Oxford Diffraction Ltd, UK, 2011.
3 CrysAlisRED, *Version 1.171.35.11 (release 16-05-2011 CrysAlis 171.NET)*, Oxford Diffraction Ltd, UK, 2011.
4 G. M. Sheldrick, SHELXT - integrated space-group and crystal-structure determination, *Acta crystallographica. Section A, Foundations and advances*, 2015, **71**, 3–8.
5 G. M. Sheldrick, Crystal structure refinement with SHELXL, *Acta crystallographica. Section C, Structural chemistry*, 2015, **71**, 3–8.
6 L. J. Farrugia, WinGX suite for small-molecule single-crystal crystallography, *J. Appl. Crystallogr.*, 1999, **32**, 837–838.
7 A. L. Spek, Single-crystal structure validation with the program PLATON, *J. Appl. Crystallogr.*, 2003, **36**, 7–13.
8 SCALE3 ABSPACK, *An Oxford Diffraction Program*, Oxford Diffraction Ltd, UK, 2005.
9 M. J. Frisch, G. W. Trucks, H. B. Schlegel, G. E. Scuseria, M. A. Robb, J. R. Cheeseman, G. Scalmani, V. Barone, G. A. Petersson, H. Nakatsuji, X. Li, M. Caricato, A. V. Marenich, J. Bloino, B. G. Janesko, R. Gomperts, B. Mennucci, H. P. Hratchian, J. V. Ortiz, A. F. Izmaylov, J. L. Sonnenberg, Williams, F. Ding, F. Lipparini, F. Egidi, J. Goings, B. Peng, A. Petrone, T. Henderson, D. Ranasinghe, V. G. Zakrzewski, J. Gao, N. Rega, G. Zheng, W. Liang, M. Hada, M. Ehara, K. Toyota, R. Fukuda, J. Hasegawa, M. Ishida, T. Nakajima, Y. Honda, O. Kitao, H. Nakai, T. Vreven, K. Throssell, J. A. Montgomery Jr., J. E. Peralta, F. Ogliaro, M. J. Bearpark, J. J. Heyd, E. N. Brothers, K. N. Kudin, V. N. Staroverov, T. A. Keith, R. Kobayashi, J. Normand, K. Raghavachari, A. P. Rendell, J. C. Burant, S. S. Iyengar, J. Tomasi, M. Cossi, J. M. Millam, M. Klene, C. Adamo, R. Cammi, J. W. Ochterski, R. L. Martin, K. Morokuma, O. Farkas, J. B. Foresman and D. J. Fox, *Gaussian 16 Rev. C.01*, Wallingford, CT, 2016.
10 Roy Dennington, Todd A. Keith and John M. Millam, *GaussView 6*, Semichem Inc., Shawnee Mission KS, 2019.
11 Mestrelab Research S.L., *MestReNova*, Santiago de Compostela, Spain, 2019.
12 A. F. Baxter, J. Schaab, K. O. Christe and R. Haiges, Perfluoroalcohols: The Preparation and Crystal Structures of Heptafluorocyclobutanol and Hexafluorocyclobutane-1,1-diol, *Angew. Chem. Int. Ed. Engl.*, 2018, **57**, 8174–8177.
